# Dynamic S-palmitoylation of glioma-associated oncogene 1 regulates the incidence and progression of Sonic Hedgehog medulloblastoma

**DOI:** 10.1038/s41419-026-09051-y

**Published:** 2026-06-29

**Authors:** Tingyu Shen, Hangyang Bao, Jirong Wang, Xing Ji, Weizhong Gu, Chao Tang, Qiangqiang He, Jiahao Luo, Dan Tan, Jiali Yao, Chengyun Xu, Ying Gong, Ximei Wu, Shi-Hong Zhang, Ling-Hui Zeng

**Affiliations:** 1 Department of Pharmacology, Zhejiang University School of Medicine, Hangzhou, China; 2 Shulan International Medical College, Zhejiang Shuren University, Hangzhou, China; 3 College of Pharmaceutical Sciences, Zhejiang University, Hangzhou, China; 4 Zhejiang Provincial Key Lab of Geriatrics, Zhejiang Hospital, Hangzhou, China; 5 Key Laboratory of Novel Targets and Drug Study for Neural Repair of Zhejiang Province, Hangzhou City University School of Medicine, Hangzhou, China; 6 National Clinical Research Center for Child Health, The Children’s Hospital of Zhejiang University School of Medicine, Hangzhou, China; 7 Department of Pharmacy, Hangzhou First People’s Hospital Affiliated of Westlake University School of Medicine, Hangzhou, China; 8 Department of Anesthesiology of the Second Affiliated Hospital, Zhejiang University School of Medicine, Hangzhou, China

**Keywords:** CNS cancer, Ubiquitylation, Paediatric cancer, Transcription factors

## Abstract

Aberrant activation of the Sonic Hedgehog (SHH)-glioma-associated oncogene 1 (GLI1) signaling pathway is a determinant driver of basal cell carcinoma (BCC) and SHH-subgroup medulloblastoma (MB_SHH_). Here, we demonstrate the role of S-palmitoylation and depalmitoylation of GLI1 in the regulation of incidence and progression of MB_SHH_. High expression of acyltransferase ZDHHC13 in MB_SHH_ induces S-palmitoylation of human GLI1 at Cys1034, stabilizes GLI1 by preventing its ubiquitin-dependent proteasomal degradation, and in turn activates this signaling pathway to promote the progression of MB_SHH_. Conversely, the deacylase ABHD17A depalmitoylates GLI1 at Cys1034, destabilizes GLI1, and in turn inactivates this signaling pathway. As a result, conditional knockout of *Zdhhc13* in cerebellar granule neuron precursors (GNPs) significantly alleviates the severity of MB_SHH_ induced by constitutively active Smoothened (*Smo-M2*) overexpression and markedly extends overall survival, whereas knockout of *Abhd17a* increases the incidence of MB_SHH_ induced by *Patched-1* ablation, rather than its progression. Together, these findings uncover the dynamic regulation of GLI1 palmitoylation and depalmitoylation by the ZDHHC13 and ABHD17A and subsequent GLI1 stabilization and destabilization as a hitherto uncharacterized mechanism controlling SHH/GLI1 signaling and may provide additional targets for therapeutic intervention of MB_SHH_.

**Figure:**
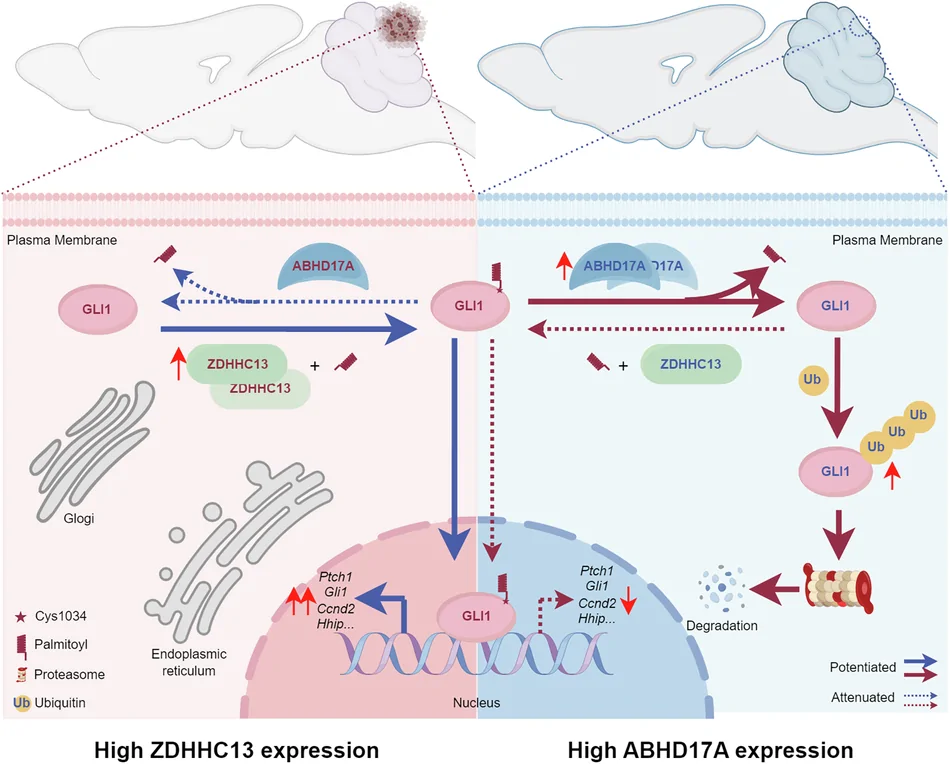

## Introduction

Medulloblastoma (MB) is the most common malignant pediatric brain tumor, accounting for approximately 20% of all childhood central nervous system neoplasms, and is molecularly classified into four subgroups: Wingless-type MMTV integration site family (WNT), Sonic Hedgehog (SHH), Group 3, and Group 4 [1]. The SHH subgroup (MB_SHH_), representing about 30% of cases, is characterized by constitutive activation of the SHH signaling pathway, due primarily to the mutations in *PTCH1*, *SMO*, or *SUFU*, or amplification of *GLI2* gene [2, 3]. Current multimodal treatments, including craniospinal irradiation and cytotoxic chemotherapy, often lead to severe neurocognitive sequelae [4], underscoring the urgent need for molecularly targeted therapies. Although FDA-approved SMO inhibitors such as vismodegib and sonidegib are effective in basal cell carcinoma (BCC) [5, 6], their utility in MB_SHH_ is limited by the development of primary and acquired resistance, driven by *SMO* mutations that impair drug binding, copy number alterations in *SUFU* and *GLI2*, and intra-tumor heterogeneity [7].

SHH signaling pathway is a highly conserved developmental regulator from *Drosophila* to mammals, governing embryonic patterning and tissue homeostasis through precise spatiotemporal regulation [8]. In mammals, this pathway is tightly coupled to the primary cilium, a microtubule-based organelle that serves as a signaling hub [9]. In the absence of SHH ligand, Patched1 (PTCH1) localizes to the cilium and suppresses Smoothened (SMO) activity, while GLI2 and GLI3 undergo phosphorylation by protein kinase A (PKA), glycogen synthase kinase 3β (GSK3β), and casein kinase 1 (CK1), promoting their proteasome-mediated processing into transcriptional repressors [10–12]. Upon SHH binding, PTCH1 exits the cilium and relieves SMO inhibition. Activated SMO then accumulates in the cilium and promotes dissociation of GLI- Suppressor of Fused (SUFU) complexes, allowing full-length GLI2/GLI3 to translocate into the nucleus as transcriptional activators, upregulating target genes including *Ptch1*, *Gli1*, and hedgehog-interacting protein (*Hhip*) [10–12].

The mammalian GLI family consists of three nuclear effectors-GLI1, GLI2, and GLI3-each exhibiting distinct transcriptional properties. GLI1 functions exclusively as a transcriptional activator, GLI2 primarily acts as an activator while retaining weak repressive capacity, and GLI3 serves predominantly as a transcriptional repressor [13]. Despite its dispensability for embryogenesis [14], GLI1 is critically involved in cell proliferation and apoptosis [15]. Clinically, oncogenic GLI1 mutations are frequently observed in multiple tumor types, particularly in MB and BCC [16, 17]. Unlike GLI2 and GLI3, GLI1 is not post-translationally processed into a truncated form. Instead, its stability, subcellular localization, and transcriptional activity are precisely controlled by multiple post‑translational modifications. For example, adenosine 5′-monophosphate-activated protein kinase (AMPK) phosphorylates GLI1 at Ser102, Ser408, and Thr1074 [18, 19], while p38α phosphorylates GLI1 at Ser937 [20]; both modifications promote GLI1 degradation. Several E3 ubiquitin ligases, including Itchy E3 ubiquitin protein ligase (ITCH) [21], p300/CBP-associated factor (PCAF) [22, 23], Tripartite motif-containing protein 32 (Trim32) [24], and SMAD-specific E3 ubiquitin protein ligase 1/2 (SMURF1/2) [25], facilitate GLI1 ubiquitination and subsequent proteasomal degradation. Conversely, the deubiquitinase ubiquitin-specific peptidase 48 (USP48) stabilizes GLI1 by removing its ubiquitin chains [26]. In addition, SUMOylation modulates the nuclear trafficking and transcriptional activity of GLI1 [27], and deacetylation enhances the transactivation potential of GLI1 and GLI2, thereby promoting the expression of downstream target genes [28].

Protein S-palmitoylation is a reversible post-translational modification catalyzed by ZDHHC acyltransferases [29] and reversed by depalmitoylases, including palmitoyl-protein thioesterase 1 (PPT1), acyl-protein thioesterases 1/2 (APT1/2), as well as alpha/beta hydrolase domain-containing protein 10 and 17A/B/C (ABHD10, ABHD17A/B/C) [30]. This modification plays a critical role in regulating protein trafficking, activity, stability, and protein-protein interactions [29]. Notably, S-palmitoylation engages in dynamic crosstalk with other post-translational modifications-such as phosphorylation [31], methylation, acetylation [32], ubiquitination [33–35], and succinylation [36]-forming complex signaling networks. Although S-palmitoylation has been identified as a key regulatory modification in numerous mammalian proteins [37, 38], it remains unknown whether GLI family members undergo this lipid modification.

The present study uncovers a novel mechanism governing GLI1 stability and transactivity, in which ZDHHC13 S-palmitoylates GLI1 at Cys1034 to prevent its ubiquitin-dependent proteasomal degradation, whereas ABHD17A acts as a deacylase to reverse this modification. As a result, genetic ablation of *Zdhhc13* in cerebellar GNPs significantly suppressed MB_SHH_ progression and extended the survival of mice bearing a constitutively active form of SMO-induced MB_SHH_. In contrast, ablation of *Abhd17a* increased the incidence of MB_SHH_ without affecting its progression in *Ptch1*^*+*^^*/−*^ mice.

## Results

### ZDHHC13 expression is significantly upregulated in human MB_SHH_

**Fig. 1:**
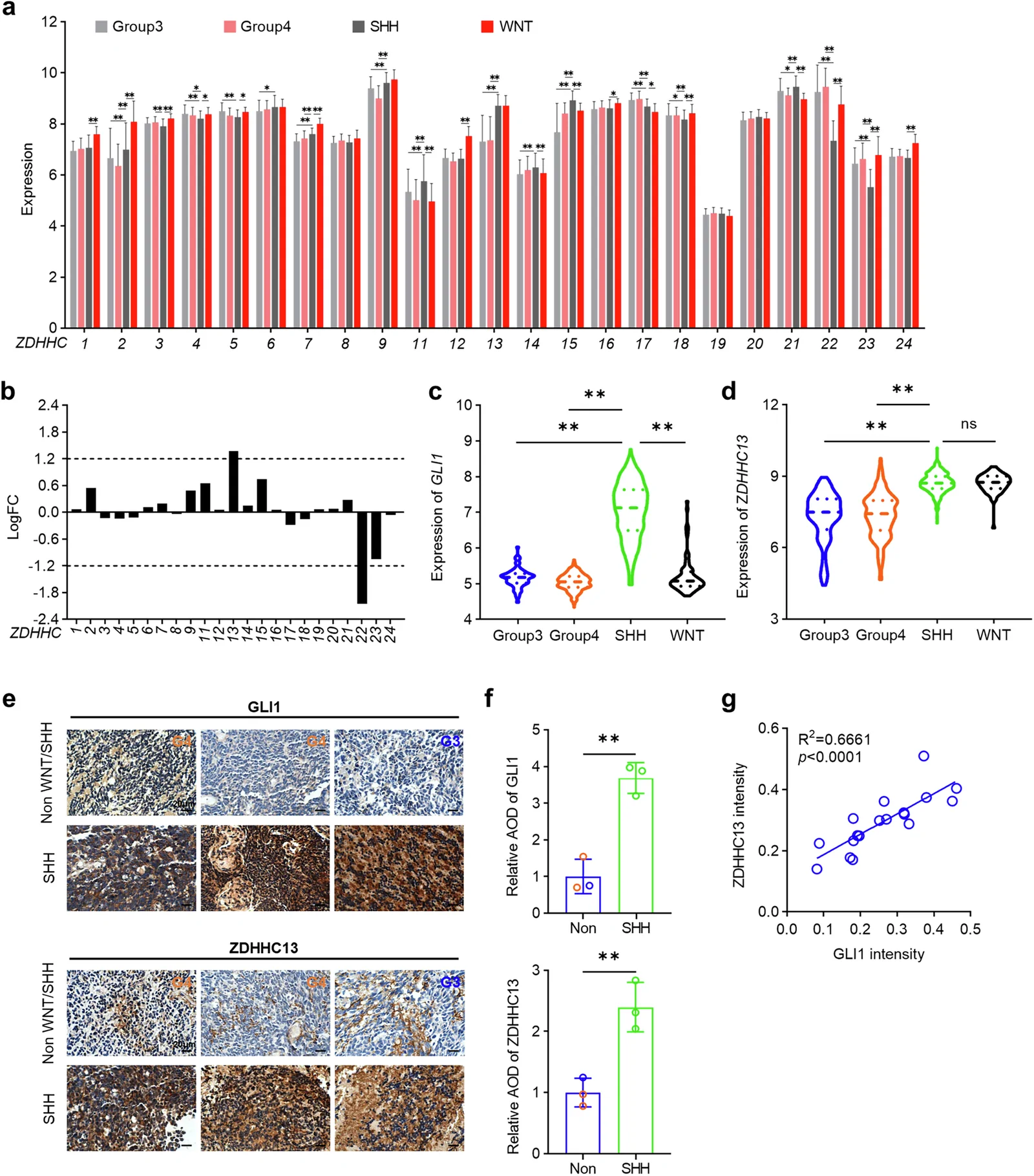
ZDHHC13 expression is significantly upregulated in human MB_SHH_. **a** Expression profiling of 23 *DHHC* genes in four subgroups of human MB. Data were obtained from the GEO dataset GSE85217 (Group3, *n* = 144; Group4, *n* = 326; SHH, *n* = 223; WNT, *n* = 70). **b** Differentially expressed *DHHC* genes in MB_SHH_ were identified using a threshold of |Log₂FC| ≥ 1.2 (fold change ≥ 2.3) compared to non-WNT/SHH subgroups. **c**, **d** Expression of *GLI1* and *ZDHHC13* across the four MB subgroups from RNA-seq data. **e**, **f** Immunohistochemistry staining and quantification of GLI1 and ZDHHC13 protein expression in human MB sections (*n* = 3). Bar=20 μm. Data points for Group 3, Group 4, and SHH subgroups are shown in blue, orange, and green, respectively. **g** Pearson’s correlation coefficient analysis between GLI1 and ZDHHC13 (*n* = 18). Mean ± SD; one-way ANOVA with Tukey’s multiple comparison test (**a**, **c**, **d**), Student’s *t*-test (**f**), or Pearson correlation coefficient test (**g**); ns: no significance, ^*^p < 0.05, ^**^p < 0.01.

To investigate the functional significance of protein palmitoylation in MB_SHH_, we first performed comprehensive transcriptomic profiling using a large-scale RNA-seq dataset that encompasses human medulloblastoma clinical specimens from the GEO database (GSE85217, *n* = 763). Systematic assessment of 23 mammalian DHHC palmitoyltransferase family members revealed distinct expression patterns across four molecular subgroups (Fig. 1a). The WNT subgroup originates from the lower rhombic lip and dorsal brainstem, whereas SHH, Group 3, and Group 4 subgroups all derive from the upper rhombic lip [39, 40], and extensive crosstalk occurs between WNT and SHH [41]. Given that Group 3 and Group 4 lack defining pathway drivers and are clinically combined as non-WNT/SHH [42], we used this combined group as a control to specifically identify *ZDHHC* genes associated with the SHH subgroup. Comparative analysis identified significant dysregulation of multiple DHHC enzymes in MB_SHH_, as compared to in non-WNT/SHH (Group 3 and Group 4) subgroups. Among these DHHC enzymes, *ZDHHC13* emerged as the most prominently upregulated gene (|LogFC| ≥ 1.2, fold change ≥ 2.3), whereas *ZDHHC22* showed marked downregulation (Fig. 1b). Given the established role of aberrant activation of SHH pathway in MB_SHH_, the selective overexpression of *ZDHHC13* suggests its potential involvement in the tumorigenesis of this malignancy.

Consistent with the aberrant activation of SHH pathway in MB_SHH_, transcript levels of *GLI1* were significantly elevated in MB_SHH_, as compared to in Group 3/4 and WNT subgroups (Fig. 1c). Parallel to the *GLI1* expression pattern, *ZDHHC13* expression showed the consistent upregulation in SHH and WNT subgroups versus in Group 3/4, despite no significant difference detected between SHH and WNT subgroups (Fig. 1d). The absence of a significant difference between the SHH and WNT subgroups may be explained by the known interplay between the WNT and SHH signaling pathways [41].

To validate our transcriptional findings at the protein level, we performed immunohistochemistry staining on human medulloblastoma tissues. Quantitative analysis revealed that the expression of GLI1 and ZDHHC13 proteins in the MB_SHH_ subgroup was increased by approximately 2.7- and 1.4-fold, as compared to the Group 3/4 subgroups, respectively (Fig. 1e, f). Correlation analysis further revealed a significant positive correlation between ZDHHC13 and GLI1 protein levels in human MB_SHH_ (*R*² = 0.6661, *p* < 0.0001) (Fig. 1g). Thus, high expression of ZDHHC13 in MB_SHH_ may have some biological relevance to MB_SHH_ tumorigenesis.

### 2-BP inactivates SHH signaling by inhibiting the S-palmitoylation of GLI1

**Fig. 2:**
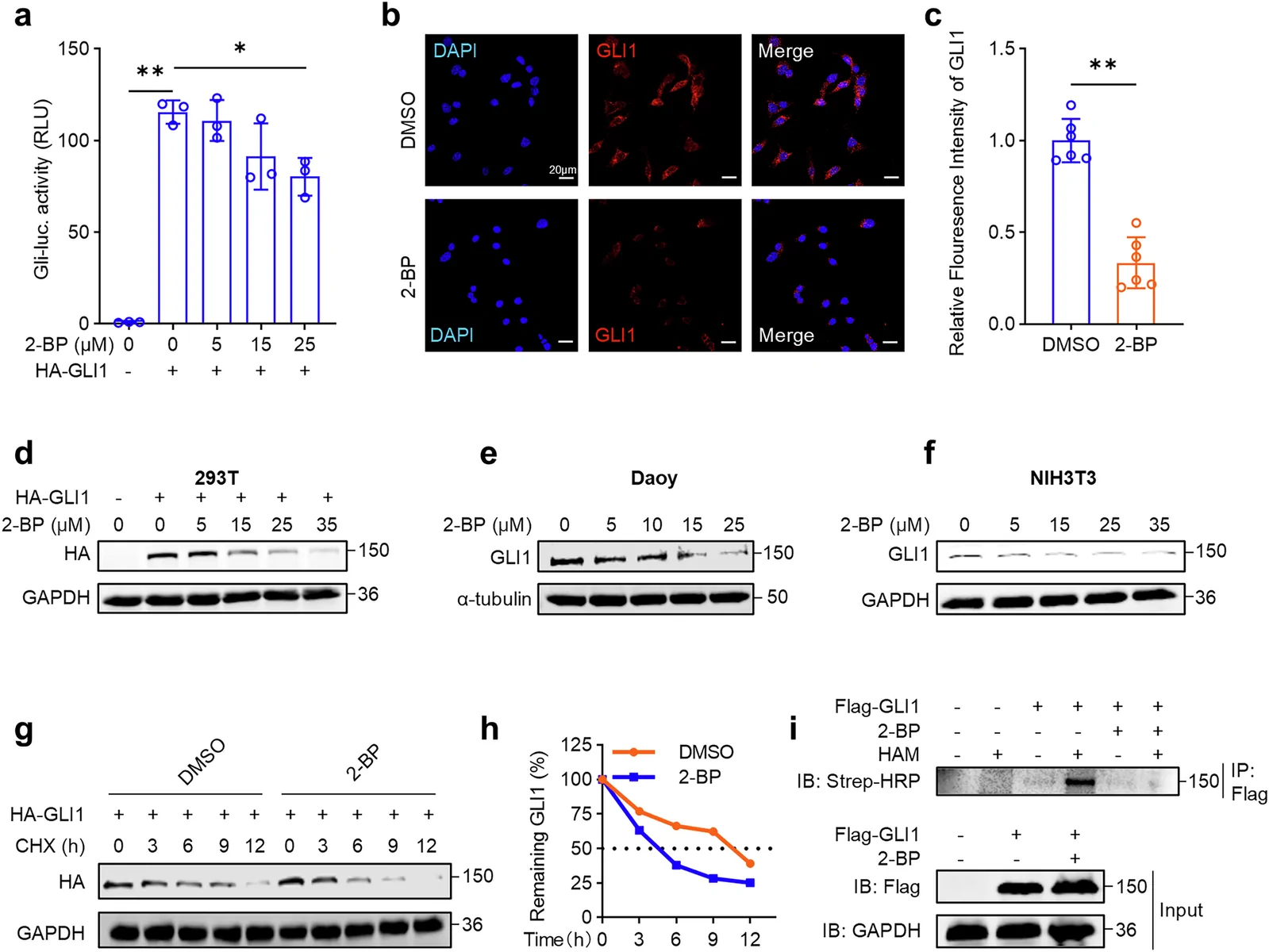
2-BP inactivates SHH signaling by inhibiting the S-palmitoylation of GLI1. **a** Gli-luciferase reporter assays in HEK293T cells co-transfected with HA-GLI1, Gli-luciferase and Renilla constructs, followed by treatment with the indicated concentrations of 2-BP for 24 h (*n* = 3). **b**, **c** Immunofluorescence staining and quantification of endogenous GLI1 in NIH3T3 cells treated with 25 μM 2-BP for 24 h (*n* = 6). Bar=20 μm. **d**–**f** Western analyzes of exogenous GLI1 protein in HA-GLI1-expressing HEK293T cells or endogenous GLI1 protein in Daoy and NIH3T3 cells, following treatment with 2-BP for 24 h at the indicated concentrations. **g**, **h** To assess the half-life of GLI1, HEK293T cells expressing HA-GLI1 were pretreated with or without 25 μM 2-BP prior to cycloheximide (100 μM) treatment for the indicated times. **i** The impact of 2-BP on GLI1 palmitoylation was assessed by the ABE assay. Mean ± SD; one-way ANOVA with Tukey’s multiple comparison test (**a**) or Student’s *t*-test (**c**); ^*^p < 0.05, ^**^p < 0.01.

To delineate the functional relationship between palmitoylation and SHH signaling pathway, we assessed the role of pan-palmitoyl acyltransferase inhibitor 2-bromopalmitate (2-BP) in the regulation of this signaling event. We first performed a dual-luciferase reporter assay in HEK293T cells co-transfected with HA-GLI1, Gli-luciferase, and Renilla constructs. GLI1 overexpression induced a 115-fold increase in luciferase activity, 2-BP treatments decreased the GLI1-induced luciferase activity in a dose-dependent manner, and 25 μM 2-BP, to the fullest extent, negated this activity by approximately 30% (Fig. 2a). Given the importance of GLI1 nuclear localization in SHH signaling, we next performed immunofluorescence analysis in SHH-responsive NIH3T3 cells. Quantitative imaging showed that 2-BP at 25 μM robustly reduced GLI1-derived fluorescence intensity in both cytoplasmic and nuclear compartments (Fig. 2b, c), implying the potential effect of 2-BP on protein stability rather than on subcellular redistribution. To test this hypothesis, we conducted Western blotting analyzes across multiple cellular models. In 293T cells transfected with HA-GLI1 construct, 2-BP reduced HA-GLI1 levels in dose- and time-dependent manner (Fig. 2d, and Supplementary Fig. 1a). This effect was conserved in human Daoy medulloblastoma and mouse NIH3T3 fibroblasts cells (Fig. 2e, f, and Supplementary Fig. 1a), suggesting that 2-BP negated GLI1 protein levels in a cell type-independent manner. Notably, 2-BP specifically targeted GLI1 without affecting full-length GLI2 or GLI3 (Supplementary Fig. 1b), underscoring the selective role of 2-BP on GLI transcriptional factors.

To explore the mechanism underlying the downregulation of GLI1 expression, we assessed transcriptional levels of *GLI1* gene. Quantitative reverse transcription polymerase chain reaction (qRT-PCR) analysis showed that *GLI1* mRNA levels were unchanged within 12 h post treatments of 2-BP (Supplementary Fig. 1d), even though GLI1 protein levels were markedly reduced within 6 h post treatments (Supplementary Fig. 1a). Transcriptional blockade by actinomycin D further confirmed the 2-BP’s effects on GLI1 protein levels (Supplementary Fig. 1c). Subsequent cycloheximide chase experiments consistently demonstrated that 2-BP treatments significantly shortened the protein half-life of GLI1 (10.5 h versus 4.5 h) (Fig. 2g, h), indicating that 2-BP accelerated degradation of GLI1 protein. To test whether GLI1 is a direct substrate of S-palmitoylation, we performed the acyl-biotin exchange (ABE) assay, which is the most widely used method for detecting protein S-palmitoylation. The results indicated that GLI1 protein was constitutively palmitoylated, and this effect was apparently abolished after 2-BP treatment (Fig. 2i). Thus, S-palmitoylation of GLI1 acts to stabilize the protein.

### ZDHHC13 palmitoylates and stabilizes GLI1 to activate SHH signaling

**Fig. 3:**
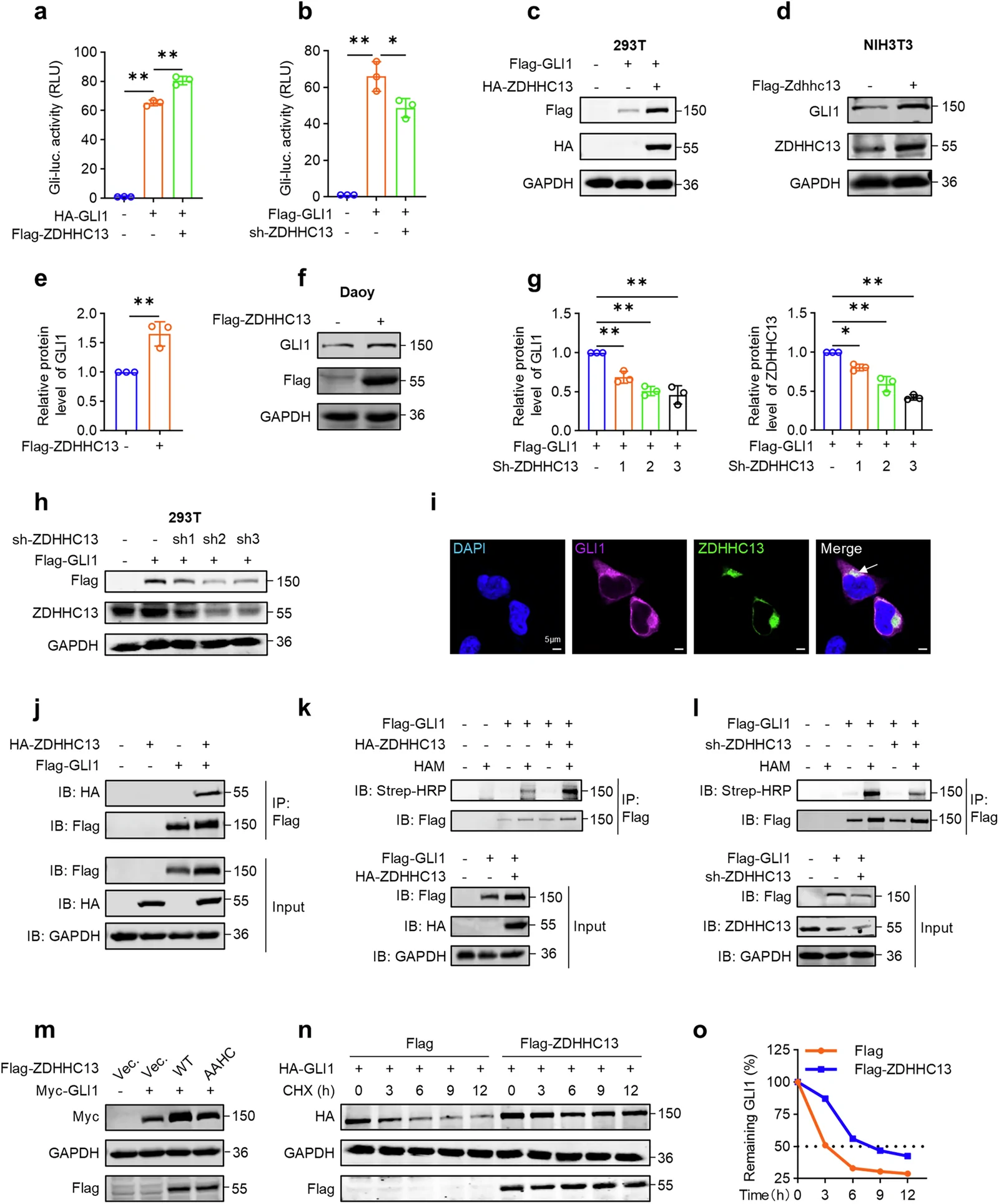
ZDHHC13 palmitoylates and stabilizes GLI1 to activate SHH signaling. **a**, **b** Gli-luciferase reporter assays in HEK293T cells co-transfected with Gli-luciferase, Renilla, and HA- or Flag-GLI1, combined with either Flag-ZDHHC13 or sh-ZDHHC13 (for 48 h and 72 h, respectively). *n* = 3. **c**, **d** Western analyzes of exogenous GLI1 protein in Flag-GLI1-expressing HEK293T cells or endogenous GLI1 protein in NIH3T3 cells following ZDHHC13 overexpression. **e**, **f** Western analyzes of endogenous GLI1 in Daoy cells following lentiviral expression of Flag-ZDHHC13, and quantification of relative GLI1 protein levels (normalized to control). *n* = 3. **g**, **h** Western analyzes of exogenous GLI1 protein in HEK293T cells co-transfected with Flag-GLI1 and different sh-ZDHHC13 constructs, with densitometric quantification of exogenous GLI1 and endogenous ZDHHC13 protein levels (normalized to control). *n* = 3. **i** Immunofluorescence analysis of co-localization of GLI1 with ZDHHC13 in HEK293T transfected with HA-ZDHHC13 and pLVX-GLI1-mcherry constructs. Bar=5 μm. **j** Co-immunoprecipitation and western analyzes in HEK293T cells transfected with HA-ZDHHC13 and Flag-GLI1 constructs. **k**, **l** ABE assays in HEK293T cells co-transfected with Flag-GLI1 and either HA-ZDHHC13 (48 h) or sh-ZDHHC13 (72 h). **m** Western analyzes of GLI1 protein in HEK293T cells co-transfected with Myc-GLI1 together with either WT or AAHC mutant Flag-ZDHHC13 for 48 h. **n**, **o** To compare the half-life of GLI1, HEK293T cells were transfected to express HA-GLI1 either alone or in combination with Flag-ZDHHC13, followed by treatment with cycloheximide (100 μM) for the indicated times. Mean ± SD; one-way ANOVA with Tukey’s multiple comparison test (**a**, **b**, **g**) or Student’s *t*-test (**e**); ^*^p < 0.05, ^**^p < 0.01.

The coincident upregulation of ZDHHC13 and GLI1 protein expression in human MB_SHH_ (Fig. 1) and stabilization of GLI1 protein after palmitoylation (Fig. 2) prompt us to investigate whether ZDHHC13 serves as a predominant acyltransferase to palmitoylate GLI1. Functionally, ZDHHC13 overexpression robustly enhanced GLI1-induced Gli-luciferase activities (Fig. 3a), whereas ZDHHC13 knockdown by its shRNA significantly suppressed reporter activities (Fig. 3b, and Supplementary Fig. 2a). Next, we assessed the effect of ZDHHC13 on GLI1 protein levels. Overexpression of ZDHHC13 increased both exogenous and endogenous GLI1 protein levels in HEK293T, NIH3T3 or Daoy cells, respectively (Fig. 3c–f, and Supplementary Fig. 2b), whereas ZDHHC13 knockdown conversely reduced both exogenous and endogenous GLI1 levels (Fig. 3g, h, and Supplementary Fig. 2c).

Immunofluorescence staining revealed the partial colocalization between perinuclear ZDHHC13 (consistent with Golgi localization) and cytoplasmic GLI1 (Fig. 3i). This physical interaction was further confirmed by the co-immunoprecipitation experiments, showing that an immunocomplex precipitated by a Flag antibody contained abundant HA-ZDHHC13 in HEK293T cells transiently expressing HA-ZDHHC13 and Flag-GLI1 (Fig. 3j). To determine whether ZDHHC13 directly catalyzes GLI1 palmitoylation, we performed ABE assays. ZDHHC13 overexpression markedly enhanced GLI1 palmitoylation levels (Fig. 3k), whereas its knockdown decreased the modification levels of GLI1 (Fig. 3l). In contrast, a catalytically dead ZDHHC13 (AAHC) mutant [43] showed a reduced ability to elevate GLI1 protein compared to wild-type ZDHHC13 (Fig. 3m), confirming the importance of its enzymatic activity. We next assessed whether ZDHHC13 regulates GLI1 at the post-translational level. Actinomycin D pretreatment did not prevent ZDHHC13-induced GLI1 accumulation (Supplementary Fig. 2d), and cycloheximide chase assays showed that ZDHHC13 overexpression significantly prolonged the half-life of GLI1 (3 h to 8 h) (Fig. 3n, o). These results support a model in which ZDHHC13 palmitoylates and, in turn, stabilizes GLI1.

To evaluate the function of ZDHHC13 in vivo, we generated GNPs-specific *Zdhhc13* knockout mice by crossing *Zdhhc13*^*f/+*^ mice with *GFAP-Cre* mice. Western analyzes showed ablation of *Zdhhc13* decreased the ZDHHC13 expression by 56% and concomitantly reduced the GLI1 protein levels by approximately 46% in cerebella of *GFAP-Cre;Zdhhc13*^*f/f*^ mice as compared to those of *Zdhhc13*^*f/f*^ mice (Supplementary Fig. 2e, f). This in vivo result corresponds to our cellular findings and establishes the conserved role of ZDHHC13 in regulating GLI1 stability across models. Together, ZDHHC13 interacts with and palmitoylates GLI1 to stabilize it.

### ABHD17A depalmitoylates and destabilizes GLI1 to inactivate SHH signaling

**Fig. 4:**
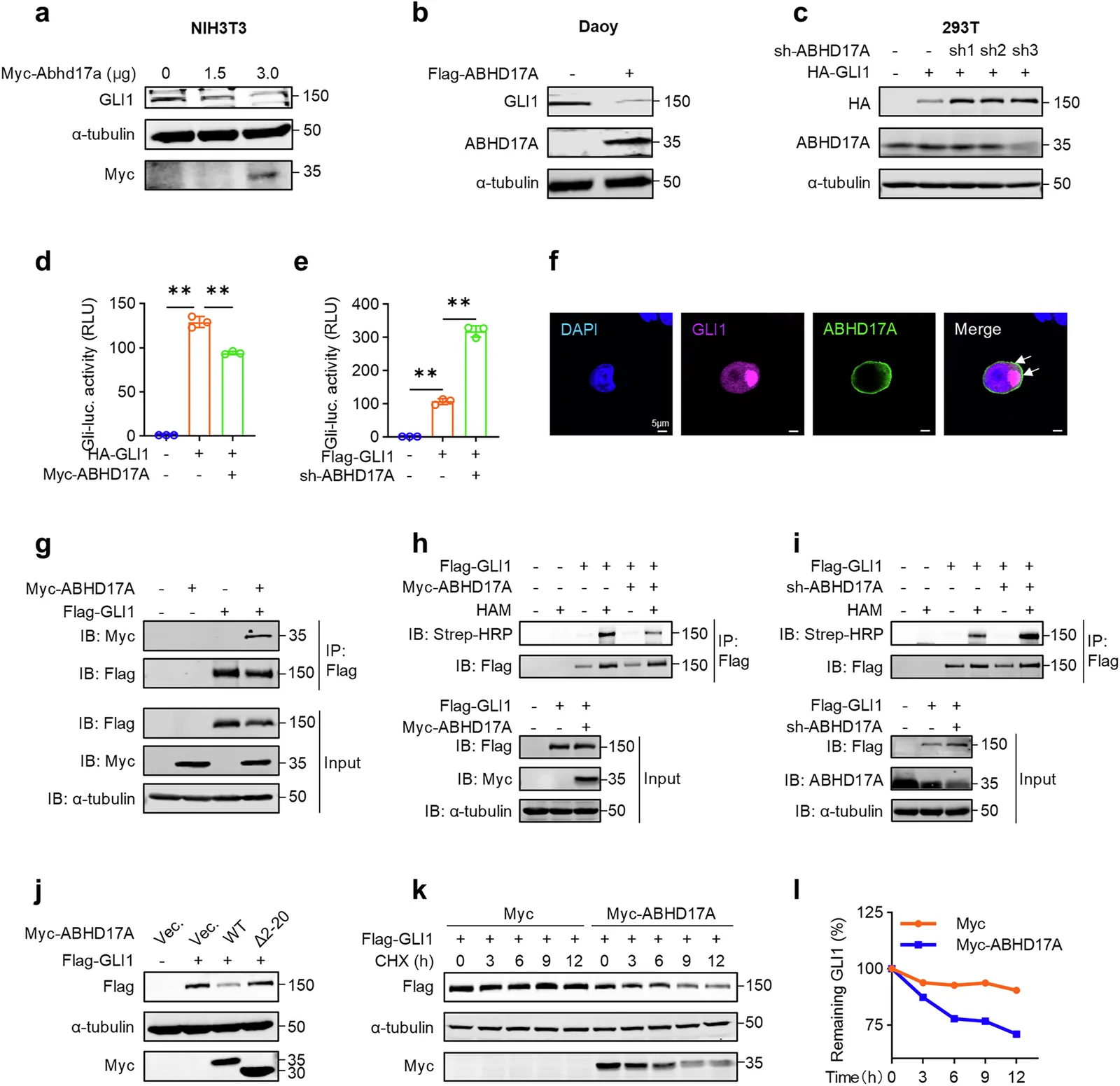
ABHD17A depalmitoylates and destabilizes GLI1 to inactivate SHH signaling. **a** Western analyzes of endogenous GLI1 protein in NIH3T3 cells transfected with 0, 1.5, or 3.0 μg Myc-Abhd17a construct. **b** Western analyzes of endogenous GLI1 in Daoy cells following lentiviral expression of Flag-ABHD17A. **c** Western analyzes of GLI1 protein in HEK293T cells following co-transfection with HA-GLI1 and various sh-ABHD17A constructs for 72 h. **d**, **e** Gli-luciferase reporter assays in HEK293T cells co-transfected with Gli-luciferase, Renilla, and HA- or Flag-GLI1, combined with either Myc-ABHD17A or sh-ABHD17A (for 48 h and 72 h, respectively). *n* = 3. **f** Immunofluorescence analysis for co-localization of GLI1 with ABHD17A in HEK293T transfected with Myc-ABHD17A and pLVX-GLI1-mcherry constructs. Bar=5 μm. **g** Co-immunoprecipitation and western analyzes in HEK293T cells transfected with Myc-ABHD17A and Flag-GLI1 constructs for 48 h. **h**, **i** ABE assays in HEK293T cells co-transfected with Flag-GLI1 and either Myc-ABHD17A (48 h) or sh-ABHD17A (72 h). **j** Western analyzes of GLI1 protein in HEK293T cells co-transfected with Flag-GLI1 together with either WT or Δ2-20 mutant Myc-ABHD17A for 48 h. **k**, **l** To compare the stability of GLI1, HEK293T cells were transfected to express Flag-GLI1 either alone or in combination with Myc-ABHD17A, followed by treatment with cycloheximide (100 μM) for the indicated times. Mean ± SD; one-way ANOVA with Tukey’s multiple comparison test (**d**, **e**); ^**^p < 0.01.

To identify the depalmitoylase responsible for GLI1 stability, we screened candidate enzymes by co-expressing Myc-GLI1 with various known depalmitoylases in HEK293T cells. The currently identified depalmitoylases primarily include PPT1, APT1, APT2, ABHD10, and members of ABHD17 family. We first excluded the PPT1 and ABHD10 according to their subcellular localizations: PPT1 and ABHD10 are predominantly lysosomal and mitochondrial proteins, respectively, whereas GLI1 localizes primarily to the cytoplasm and nucleus. Among the remaining enzymes, ABHD17A, instead of other enzymes, specifically reduced GLI1 protein levels (Supplementary Fig. 3a, b). This effect was dose-dependent in NIH3T3 cells and conserved in Daoy medulloblastoma cells (Fig. 4a, b, and Supplementary Fig. 3c). Conversely, ABHD17A knockdown using validated shRNAs increased both exogenous and endogenous GLI1 levels (Fig. 4c, and Supplementary Fig. 3d, e). Functionally, ABHD17A overexpression significantly attenuated GLI1-induced Gli-luciferase activities, whereas its knockdown robustly enhanced GLI1-driven luciferase activities (Fig. 4d, e). Thus, ABHD17A mirrors the effects of 2-BP to destabilize GLI1 and, in turn, inactivate SHH signaling,

Immunofluorescence staining revealed the partial colocalization between membrane-proximal ABHD17A and cytoplasmic GLI1 (Fig. 4f), consistent with the immunoprecipitation results demonstrating their physical interaction in HEK293T cells (Fig. 4g). We next examined whether ABHD17A directly catalyzes GLI1 depalmitoylation using ABE assays. ABHD17A overexpression reduced GLI1 palmitoylation, while its knockdown enhanced this modification (Fig. 4h, i). Importantly, the catalytically inactive and membrane-localization-deficient ABHD17A mutant (Δ2-20) [44] failed to reduce GLI1 levels, as compared to the wild-type ABHD17A (Fig. 4j), confirming the requirement of catalytic activity. To determine the role of ABHD17A in regulating GLI1 stability, we treated cells with actinomycin D and found that it didn’t prevent ABHD17A-mediated GLI1 reduction (Supplementary Fig. 3f), excluding the possibility that ABHD17A regulates the transcription of *GLI1* gene. Whereas cycloheximide chase assays demonstrated that ABHD17A overexpression accelerated GLI1 degradation (Fig. 4k, l).

To explore the physiological relevance of ABHD17A, we analyzed *Abhd17a* knockout (KO) mice, which exhibited extremely low ABHD17A expression at both mRNA and protein levels and significantly high GLI1 protein expression in cerebella (Supplementary Fig. 3g, h). Interestingly, *Abhd17a* mRNA expression was reduced by 50% in cerebella from *GFAP-Cre;Smo-M2* medulloblastoma mice (Supplementary Fig. 3i), suggesting its potential tumor-suppressive role. Together, ABHD17A depalmitoylates and subsequently destabilizes GLI1 to suppress SHH signaling in both cellular and physiological settings.

### Cys1034 is responsible for ZDHHC13/ABHD17A-induced human GLI1 palmitoylation/depalmitoylation

**Fig. 5:**
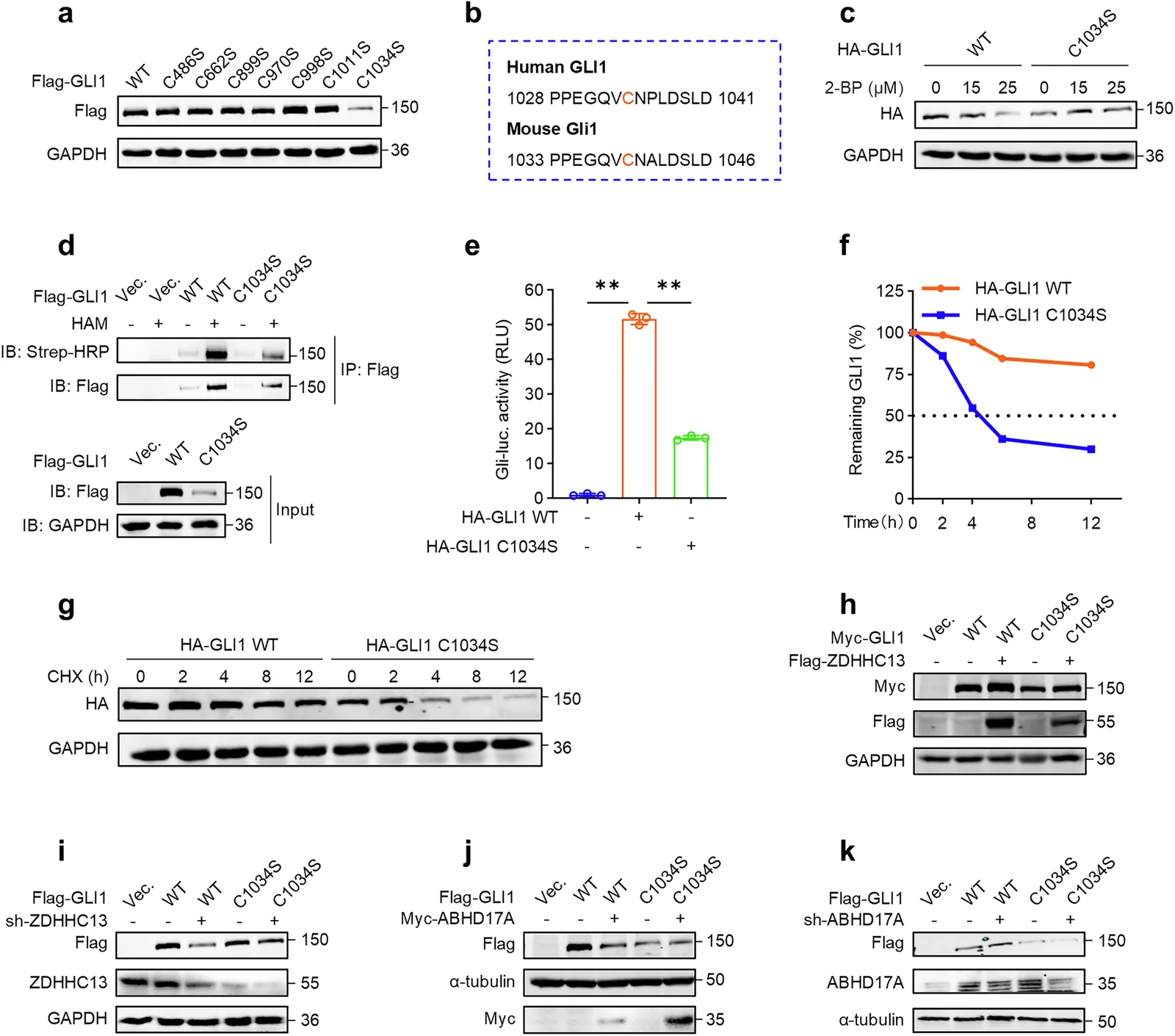
Cys1034 is responsible for ZDHHC13/ABHD17A-induced human GLI1 palmitoylation/depalmitoylation. **a** Western analyzes of Flag-tagged GLI1 mutants, in which all cysteines within segments 470-669 and 887-1106 were mutated to serines, expressed in HEK293T cells. **b** Cys1034 site of human GLI1 is conserved between humans (Cys1034) and mice (Cys1039). **c** Western analyzes of HA-GLI1 WT and C1034S mutant expressed in HEK293T cells and treated with the indicated concentrations of 2-BP for 12 h. **d** ABE assays assessing the palmitoylation of Flag-tagged GLI1 WT and C1034S mutant in HEK293T cells. **e** Gli-luciferase reporter assays analyzing the transcriptional activity of HA-tagged GLI1 WT and C1034S mutant in HEK293T cells (*n* = 3). **f**, **g** To compare the half-life of GLI1, HEK293T cells expressing HA-tagged GLI1 WT or the C1034S mutant were treated with cycloheximide (100 μM) for the indicated times. Western analyzes of exogenous GLI1 protein under the following co-expression conditions: Myc-GLI1 WT or C1034S with or without Flag-ZDHHC13 for 48 h (**h**); Flag-GLI1 WT or C1034S with or without sh-ZDHHC13 for 72 h (**i**); Flag-GLI1 WT or C1034S with or without Myc-ABHD17A for 48 h (**j**); and Flag-GLI1 WT or C1034S with or without sh-ABHD17A for 72 h (**k**). Mean ± SD; one-way ANOVA with Tukey’s multiple comparison test (**e**); ^**^p < 0.01.

We next attempted to identify the specific modification site(s) by ZDHHC13 and ABHD17A and generated a series of GLI1 truncation mutants spanning its functional domains (Δ1-234, Δ235-469, Δ470-669, Δ670-886, and Δ887-1106; Supplementary Fig. 4a). Systematic analysis revealed that deletion of two regions (aa470-669 and aa887-1106) rendered GLI1 resistant to 2-BP-induced degradation (Supplementary Fig. 4b). As palmitoylation primarily occurs on cysteine residues, we expressed GLI1 variants that harbor mutations at the seven consensus cysteine residues (Cys to Ser) within these regions individually (C486S, C662S, C899S, C970S, C998S, C1011S, C1034S) and evaluated their capacity to affect the expression of GLI1. C1034S instead of other mutations markedly reduced the basal levels of GLI1 protein, and this cysteine residue is evolutionarily conserved in mouse (Cys1039) and human (Fig. 5a, b). C1034S mutation not only significantly decreased the palmitoylation of GLI1 but also robustly abolished the effect of 25 μM 2-BP on reducing GLI1 protein (Fig. 5c, d). As a result, the C1034S mutant reduced the GLI1-induced Gli-luciferase activities by 66% (Fig. 5e), and was degraded substantially faster than wild-type GLI1 (*t*_*1/2*_: 4.1 h versus 27.7 h) (Fig. 5f, g), confirming that palmitoylation at Cys1034 is required for GLI1 stability.

To test whether the dynamic S-palmitoylation of GLI1 at Cys1034 is specifically regulated by ZDHHC13 and ABHD17A, we performed western analyzes in HEK293T cells transiently expressing either WT or C1034S mutant of GLI1. Overexpression of ZDHHC13 and ABHD17A apparently increased and decreased GLI1 levels in WT- but not in C1034S-expressing cells, respectively (Fig. 5h, j), and knockdown of ZDHHC13 and ABHD17A by their respective shRNA conversely reduced and enhanced GLI1 levels in WT- but not in C1034S-expressing cells, respectively (Fig. 5i, k). These results indicate that Cys1034 acts as a specific site of GLI1 for both ZDHHC13-mediated palmitoylation and ABHD17A-catalyzed depalmitoylation.

### Depalmitoylation promotes the ubiquitin-dependent proteasomal degradation of GLI1

**Fig. 6:**
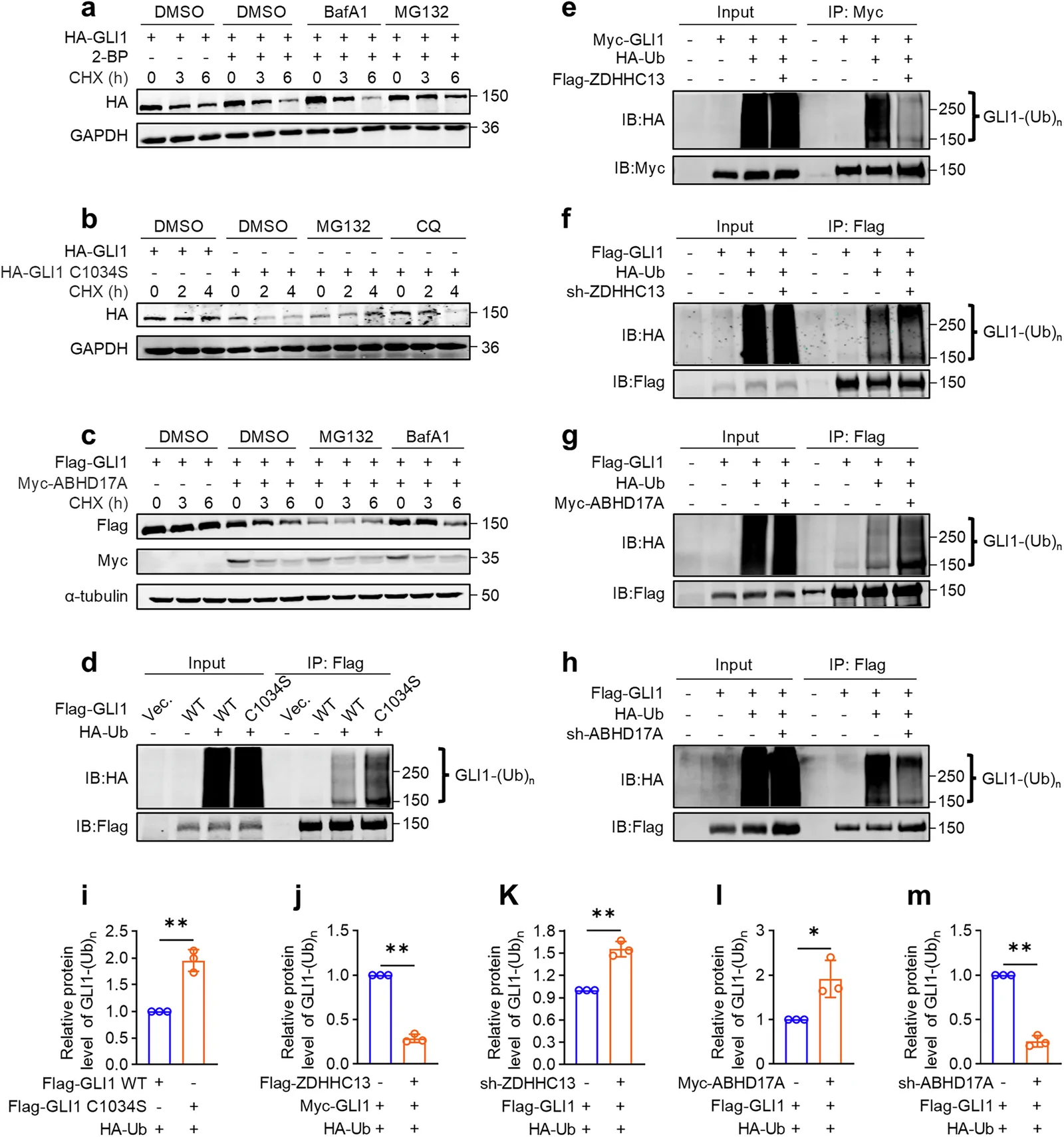
Depalmitoylation promotes the ubiquitin-dependent proteasomal degradation of GLI1. GLI1 protein stability was analyzed by cycloheximide chase in HEK293T cells under treatment with various pharmacological agents: HA-GLI1-expressing cells with 2-BP (25 μM, 24 h), BafA1 (100 nM, 6 h), or MG132 (10 μM, 12 h) (**a**); HA-GLI1 WT or C1034S mutant-expressing cells with CQ (10 μM, 24 h) or MG132 (10 μM, 12 h) (**b**); Flag-GLI1/Myc-ABHD17A-coexpressing cells with BafA1 (100 nM, 6 h), or MG132 (10 μM, 12 h) (**c**). Assessment of GLI1 ubiquitination by co-immunoprecipitation and western analyzes in HEK293T cells co-transfected with HA-ubiquitin and the following constructs: Flag-GLI1 WT or C1034S mutant (**d**); Myc-GLI1 with Flag-ZDHHC13 (**e**); Flag-GLI1 with sh-ZDHHC13 (**f**); Flag-GLI1 with Myc-ABHD17A (**g**); Flag-GLI1 with sh-ABHD17A (**h**). **i**–**m** The relative level of ubiquitinated GLI1 was quantified and normalized to the control (*n* = 3). Mean ± SD; Student’s *t*-test; ^*^p < 0.05, ^**^p < 0.01.

Protein degradation occurs mainly through three pathways: the ubiquitin-proteasome system (UPS), the autophagy-lysosome pathway (ALP), and the endosome-lysosome pathway (ELP). To determine how depalmitoylated GLI1 is degraded, we inhibited each pathway by using MG132 (proteasome inhibitor), Bafilomycin A1 (BafA1, autophagy inhibitor), and Chloroquine (CQ, lysosome inhibitor), respectively. Western analyzes showed MG132, but not BafA1, robustly restored the 2-BP-induced GLI1 degradation (Fig. 6a). Likewise, MG132 significantly prevented GLI1 degradation induced by either the C1034S mutation or ABHD17A overexpression, both of which mimic GLI1 depalmitoylation; however, neither BafA1 nor CQ restored GLI1 degradation under these latter conditions (Fig. 6b, c). These findings suggest that UPS acts as the primary degradation pathway for depalmitoylated GLI1 protein.

Given the emerging evidence of functional crosstalk between palmitoylation and ubiquitination, we hypothesized that GLI1 palmitoylation regulates its stability via ubiquitination and subsequent proteasomal degradation. Co-immunoprecipitation assays confirmed the polyubiquitination of GLI1 and revealed significantly more ubiquitination in C1034S mutant than in WT (Fig. 6d, i). Consistently, suppression of palmitoylation by ZDHHC13 knockdown or ABHD17A overexpression increased GLI1 ubiquitination (Fig. 6f, g, k, l); in contrast, enhancement of palmitoylation by ZDHHC13 overexpression or ABHD17A knockdown reduced GLI1 ubiquitination (Fig. 6e, h, j, m). Thus, palmitoylation at Cys1034 protects GLI1 from polyubiquitination and proteasomal degradation and thereby stabilize GLI1 to activate SHH signaling pathway.

### *Zdhhc13* loss-of-function relieves the severity of MB_SHH_ induced by *SMO-M2* overexpression in mice

**Fig. 7:**
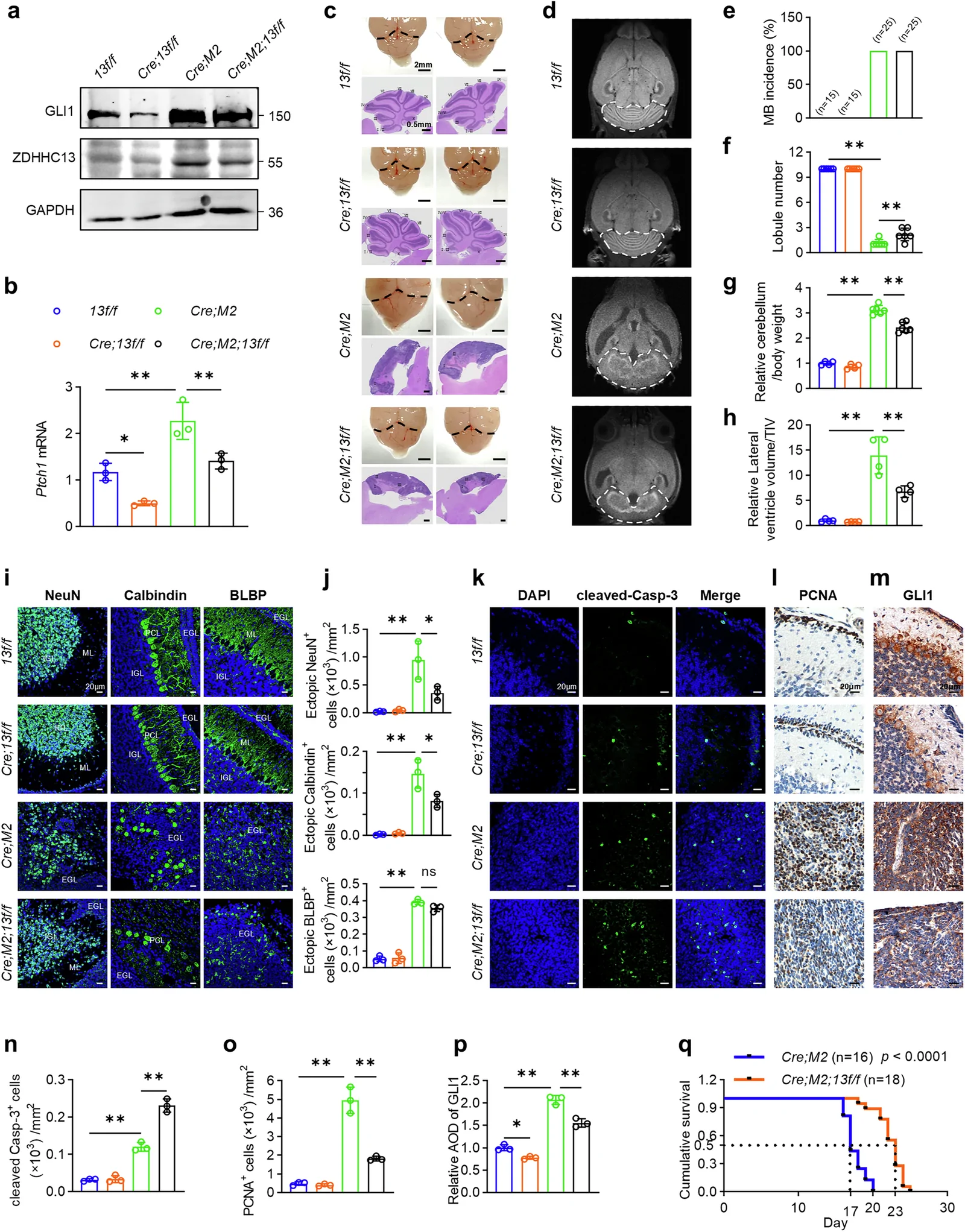
*Zdhhc13* loss-of-function relieves the severity of MB_SHH_ induced by *SMO-M2* overexpression in mice. **a**, **b** Western analyzes of GLI1 protein and qRT-PCR analysis of *Ptch1* mRNA in cerebella of *Zdhhc13*^*f/f*^, *GFAP-Cre;Zdhhc13*^*f/f*^, *GFAP-Cre;SMO-M2* and *GFAP-Cre;Zdhhc13*^*f/f*^*;SMO-M2* pups at 2 weeks of age (*n* = 3). **c** Gross appearance of the cerebellum (Bar=2 mm) and H&E staining of cerebellar paraffin sections (Bar=0.5 mm). Roman numerals label the cerebellar folia in the H&E images. **d** Manganese-enhanced horizontal T1-weighted MRI images of brains. Intraperitoneal injection of MnCl_2_ was conducted 24 h prior to MRI scanning. The cerebellum is outlined with a white dashed line. **e** Incidence of MB_SHH_ in pups at 2 weeks of age (*n* = 15 or 25). **f**, **g** The number of folia in the sagittal section of the cerebellar vermis (*n* = 6) and quantification of cerebellar weight/body weight ratio (*n* = 4 or 6). **h** Semi-automated segmentation of T1‑weighted 2D MRI sequences was performed using 3D Slicer, and the lateral ventricular‑to‑TIV ratio was quantified. TIV refers to total intracranial volume (*n* = 4). **i**, **j** Anti-NeuN, anti-Calbindin and anti-BLBP immunofluorescence labeling and quantification in the sagittal sections of the cerebellar vermis from 2-week-old pups (*n* = 3). Bar=20 μm. **k**–**p** Cleaved casepase-3 immunofluorescence, PCNA and GLI1 immunohistochemistry staining, and corresponding quantification analyzes in cerebellar sections (*n* = 3). Bar=20 μm. **q** Overall survival rates of *GFAP-Cre;Smo-M2* pups (*n* = 16) and *GFAP-Cre;Zdhhc13*^*f/f*^*;SMO-M2* pups (*n* = 18) with MB_SHH_. Median survival times from Log-rank analysis are shown in graph. Mean ± SD; one-way ANOVA with Tukey’s multiple comparison test (**b**, **f**–**h**, **j**, **n**–**p**) or log-rank test (**q**); ns: no significance, ^*^p < 0.05, ^**^p < 0.01.

MB_SHH_ originates from GNPs in the cerebellar hemispheres during early postnatal development (P1-P7) [45, 46]. GNPs undergo robust expansion within the external granular layer (EGL) under strict SHH signaling regulation. Dysregulation of this pathway disrupts normal developmental progression, promoting sustained GNPs proliferation and ultimately driving tumorigenesis [47]. Given the fact that the peak proliferative phase (P1-P7) coincides with markedly elevated cerebellar GLI1 expression [48], we performed immunofluorescence staining on P4 mouse cerebellar sections. Immunostaining revealed the substantial colocalization of ZDHHC13 with GLI1 in both the EGL and Purkinje cell layer (PCL) (Supplementary Fig. 5a, white arrows), supporting their interaction in MB_SHH_-originating GNPs. To further investigate the functional significance of ZDHHC13-mediated GLI1 palmitoylation in MB_SHH_ pathogenesis, we utilized *GFAP-Cre;Smo-M2* transgenic mice that spontaneously develop MB_SHH_, and generated 2-week-old littermates with four genotypes (*Zdhhc13*^*f/f*^, *GFAP-Cre;Zdhhc13*^*f/f*^, *GFAP-Cre;Smo-M2*, and *GFAP-Cre;Zdhhc13*^*f/f*^*;Smo-M2*). *GFAP-Cre;Smo-M2* cerebella showed significantly elevated GLI1 protein levels and *Ptch1* mRNA expression as compared to *Zdhhc13*^*f/f*^ control cerebella. Notably, conditional ablation of *Zdhhc13* substantially reduced both GLI1 protein and *Ptch1* transcript levels regardless of SMO activation status (Fig. 7a, b). These in vivo results correspond to our cellular findings and confirmed ZDHHC13 as a positive regulator of GLI1 stability and SHH signaling output.

We next analyzed the cerebellar phenotypes of *Zdhhc13*^*f/f*^, *GFAP-Cre;Zdhhc13*^*f/f*^, *GFAP-Cre;Smo-M2*, and *GFAP-Cre;Zdhhc13*^*f/f*^*;Smo-M2* littermates at 2-week-old. Though no phenotypic abnormalities were observed in *GFAP-Cre;Zdhhc13*^*f/f*^ cerebella compared to *Zdhhc13*^*f/f*^ cerebella, *GFAP-Cre;Smo-M2* mice displayed characteristic disease manifestations such as cranial protrusions, lethargy, unsteady gait, and severe hydrocephalus, and *GFAP-Cre;Zdhhc13*^*f/f*^*;Smo-M2* mice showed marked improvement in these symptoms (Fig. 7c). Quantitative assessment showed that the cerebellum-to-body weight ratios were significantly elevated in *GFAP-Cre;Smo-M2* littermates as compared to *Zdhhc13*^*f/f*^ controls, whereas this increase was significantly attenuated in *GFAP-Cre;Zdhhc13*^*f/f*^*;Smo-M2* littermates (Fig. 7g). Histopathological examination further supported tumor suppressive roles of *Zdhhc13* ablation. H&E staining for sagittal sections showed the normal cerebellar structures in *Zdhhc13*^*f/f*^ and *GFAP-Cre;Zdhhc13*^*f/f*^ littermates (0% tumor incidence), whereas *GFAP-Cre;Smo-M2* cerebella exhibited 100% tumor incidence with extensive GNPs proliferation, complete foliation loss, and enlarged cerebellar sizes. Although *GFAP-Cre;Zdhhc13*^*f/f*^*;Smo-M2* mice also showed 100% tumor incidence, pathological severity was markedly improved with partial preservation of cerebellar foliation (Fig. 7c, e, f). MEMRI findings corresponded to the pathological observations in these littermates. *Zdhhc13*^*f/f*^ and *GFAP-Cre;Zdhhc13*^*f/f*^ mice exhibited intact cerebellar foliation; *GFAP-Cre;Smo-M2* mice showed complete foliation loss and severe hydrocephalus, whereas *GFAP-Cre;Zdhhc13*^*f/f*^*;Smo-M2* mice displayed attenuated hydrocephalus. Quantitative analysis of lateral ventricle volume-to-total intracranial volume (TIV) ratio further supported these findings. Compared with *Zdhhc13*^*f/f*^ or *GFAP-Cre;Zdhhc13*^*f/f*^ mice, the lateral ventricle volume-to-TIV ratio was markedly increased in *GFAP-Cre;Smo-M2* mice. Moreover, relative to *GFAP-Cre;Smo-M2* mice, this ratio was significantly reduced in *GFAP-Cre;Zdhhc13*^*f/f*^*;Smo-M2* mice, indicating that *Zdhhc13* deficiency alleviated hydrocephalus in this genotype (Fig. 7d, h, and Supplementary Fig. 5b).

To further validate these pathological phenotypes, we performed immunofluorescence staining on P14 cerebellar sections using antibodies against NeuN, calbindin, and BLBP to identify post-mitotic granule neurons, Purkinje cells (PCs), and Bergmann glia (BGs), respectively. *Zdhhc13*^*f/f*^ and *GFAP-Cre;Zdhhc13*^*f/f*^ littermates exhibited a well-organized cerebellar architecture with NeuN^+^ granule cells (GCs) properly localized to the internal granular layer (IGL) and Calbindin^+^ PCs neatly aligned in the PCL. In contrast, *GFAP-Cre;Smo-M2* littermates exhibited severe structural disorganization characterized by ectopic NeuN^+^ cells dispersed throughout the molecular layer (ML) and disrupted PCs arrangement. Notably, genetic deletion of *Zdhhc13* in *GFAP-Cre;Zdhhc13*^*f/f*^*;Smo-M2* littermates substantially ameliorated these pathological architectures, significantly reduced ectopic granule cell localization and partially restored normal PCs distribution. BGs exhibited marked dispersion in *GFAP-Cre;Smo-M2* mice, contrasting with their organized arrangement in both *Zdhhc13*^*f/f*^ and *GFAP-Cre;Zdhhc13*^*f/f*^ controls. However, this glial disorganization was similarly observed in *GFAP-Cre;Zdhhc13*^*f/f*^*;Smo-M2* littermates without significant improvement (Fig. 7i, j). Together, these findings demonstrate that ablation of *Zdhhc13* partially restores cerebellar architecture in the *Smo-M2*-driven medulloblastoma.

We further evaluated apoptosis and proliferation in cerebellar sagittal paraffin sections using antibodies against cleaved caspase-3 and proliferating cell nuclear antigen (PCNA), respectively. Given that MB_SHH_ originates from GNPs, we quantified positive cells specifically within the EGL of the cerebellum. No significant difference in the number of cleaved caspase-3^+^ cells was observed between the cerebella of *Zdhhc13*^*f/f*^ and *GFAP-Cre;Zdhhc13*^*f/f*^ littermates. In *GFAP-Cre;Smo-M2* littermates, EGL expansion led to an increase in total GNP number, resulting in a significant ~4-fold increase in cleaved caspase-3^+^ cells compared to *Zdhhc13*^*f/f*^ controls. Moreover, *GFAP-Cre;Zdhhc13*^*f/f*^*;Smo-M2* littermates exhibited a further significant increase in cleaved caspase-3^+^ cells relative to *GFAP-Cre;Smo-M2* littermates (Fig. 7k, n). PCNA immunohistochemistry revealed a marked increase in PCNA^+^ cells in *GFAP-Cre;Smo-M2* littermates as compared to *Zdhhc13*^*f/f*^ controls, approximately tenfold higher, whereas no significant difference was detected between *Zdhhc13*^*f/f*^ and *GFAP-Cre;Zdhhc13*^*f/f*^ littermates. Notably, *GFAP-Cre;Zdhhc13*^*f/f*^*;Smo-M2* littermates showed a significant reduction in PCNA^+^ cells as compared to *GFAP-Cre;Smo-M2* littermates (Fig. 7l, o). In addition, immunohistochemistry indicated that GLI1 was predominantly localized to PCs and GNPs. Quantitative analysis demonstrated a robust increase in GLI1-derived signals in *GFAP-Cre;Smo-M2* littermates as compared to *Zdhhc13*^*f/f*^ controls, whereas ablation of *Zdhhc13* significantly reduced GLI1-derived signals, regardless of SMO activation status (Fig. 7m, p). Collectively, these results indicate that ablation of *Zdhhc13* downregulates GLI1 protein levels, attenuates SHH signaling, and suppresses tumor progression by promoting apoptosis and inhibiting proliferation.

Finally, to evaluate the impact of ablation of *Zdhhc13* on MB_SHH_ progression, we performed survival analysis in *GFAP-Cre;Smo-M2* and *GFAP-Cre;Zdhhc13*^*f/f*^*;Smo-M2* littermates. Kaplan–Meier curves demonstrated that *Zdhhc13* ablation significantly extended median survival from 17 to 23 days and overall survival from 20 to 25 days (*p* < 0.0001, log-rank test) (Fig. 7q). Thus, deletion of *Zdhhc13* attenuates MB_SHH_ progression and improves survival in the *GFAP-Cre;Smo-M2* medulloblastoma model.

### *Abhd17a* loss-of-function increases the incidence of MB_SHH_ in *Ptch1*^*+/*^^*−*^ mice

As previously demonstrated, GLI1 protein levels were significantly upregulated in the *Abhd17a* knockout (KO) cerebella as compared to wild-type (WT) controls (Supplementary Fig. 3h). Quantitative real-time PCR analysis consistently revealed that *Abhd17a* KO markedly increased mRNA expression of *Ptch1*, the target gene of SHH signaling pathway (Supplementary Fig. 6a). To determine whether *Abhd17a* KO could induce MB_SHH_ by activating SHH signaling, we performed H&E staining for cerebellar sections. At both P14 and 3 months of age, *Abhd17a* KO mice exhibited normal cerebellar foliation without trend of nodularity or tumorigenesis (Supplementary Fig. 6b, c). Moreover, immunofluorescence analysis revealed well-organized GCs, PCs, and BGs in the IGL, PCL, and ML, respectively, without differences between groups (Supplementary Fig. 6e–h). Thus, *Abhd17a* KO alone is insufficient to drive MB_SHH_ formation due primarily to a subthreshold level of SHH activation and inadequateness to induce GNPs hyperproliferation. Notably, ABHD17A exhibited a similar spatial distribution (PCL and EGL) to ZDHHC13 as well as to GLI1 at P4 (Supplementary Fig. 6d).

**Fig. 8:**
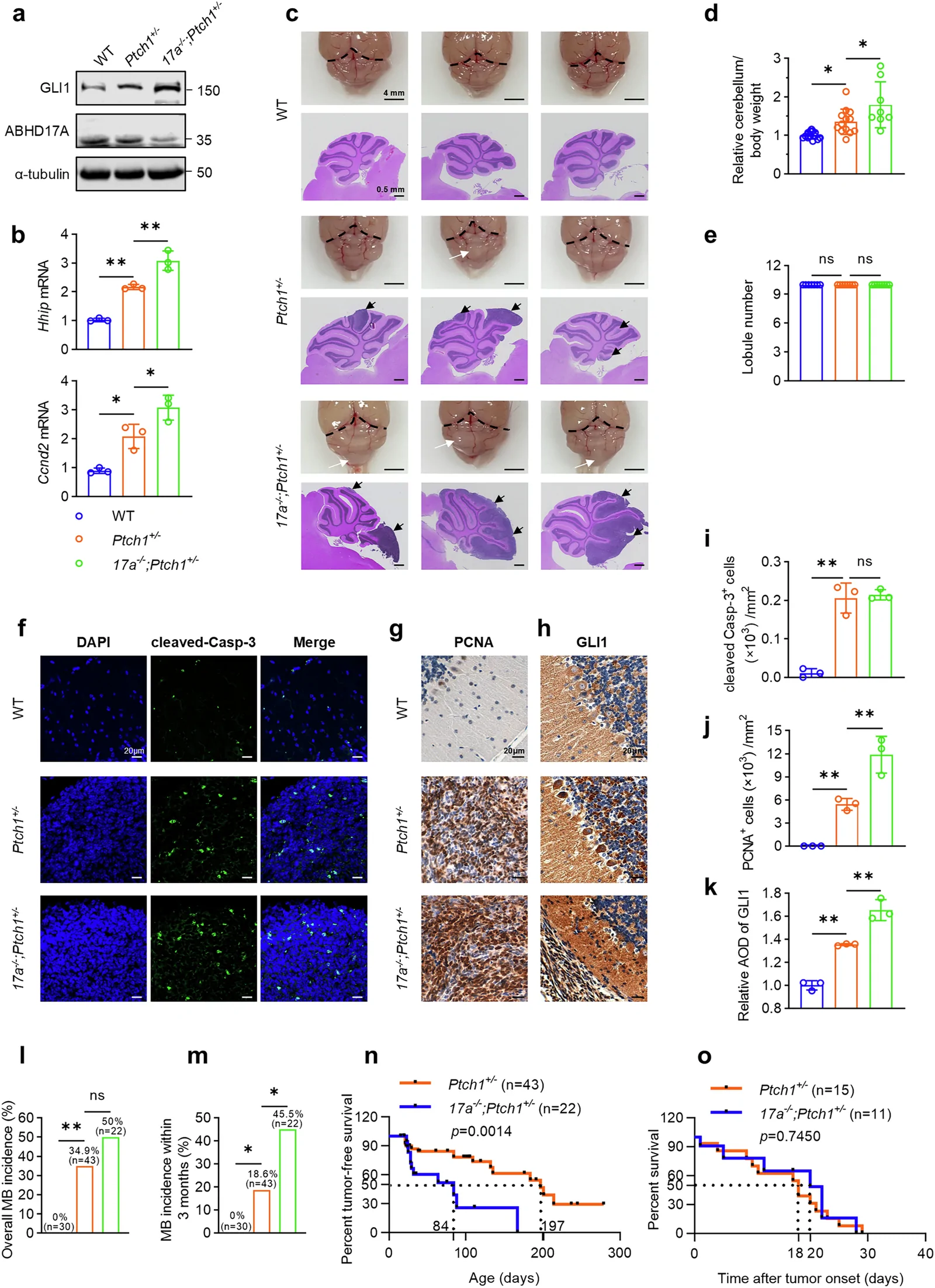
*Abhd17a* loss-of-function increases the incidence of MB_SHH_ in *Ptch1*^*+/*^^−^ mice. **a,** **b** Western analyzes of GLI1 protein and qRT-PCR analysis of SHH target genes *Hhip* and *Ccnd2* mRNAs in cerebella of WT, *Ptch1*^*+/*^^*−*^ and *Abhd17a*^*−/−*^*;Ptch1*^*+/*^^*−*^ mice at 3 months of age (*n* = 3). **c** Gross appearance of the cerebellum (Bar=4 mm) and H&E staining of cerebellar paraffin sections (Bar=0.5 mm). **d**, **e** Quantification of cerebellar weight/body weight ratio (*n* = 13 or 8) and the number of folia in the sagittal section of the cerebellar vermis (*n* = 7 or 8). **f**–**k** Cleaved casepase-3 immunofluorescence, PCNA and GLI1 immunohistochemistry staining, and corresponding quantification analyzes in cerebellar sections (*n* = 3). Bar=20 μm. **l**, **m** Overall MB incidence in WT (*n* = 30), *Ptch1*^*+/*^^*−*^ (*n* = 43) and *Abhd17a*^−/−^*;Ptch1*^*+/−*^ (*n* = 22) mice across all ages, and incidence within three months of age. **n**, **o** Tumor-free survival (time from birth to first tumor, *n* = 43 or 22) and post-tumor survival (survival after first tumor detection, *n* = 15 or 11) in *Ptch1*^*+/−*^ and *Abhd17a*^*−/−*^*;Ptch1*^*+/*^^*−*^ mice. Median survival times from Log-rank analysis are shown in graph. Mean ± SD; one-way ANOVA with Tukey’s multiple comparison test (**b**, **d**, **e**, **i**, **j**, **k**), Fisher’s exact test (**l**, **m**), or log-rank test (**n**, **o**); ns: no significance, ^*^p < 0.05, ^**^p < 0.01.

To investigate further the functional role of the depalmitoylase ABHD17A in MB_SHH_ pathogenesis, we employed *Ptch1*^+/^^−^ mice, a well-established mouse model of MB_SHH_. Heterozygous loss of *Ptch1* results in constitutive pathway activation and promotes MB_SHH_ development. *Ptch1*^+/^^−^ mice typically develop MB_SHH_ with 15-20% incidence between 3 and 6 months of age, whereas *Ptch1*^−/−^ homozygotes are embryonically lethal [49]. By crossing *Ptch1*^+/^^−^ with *Abhd17a*^+/^^−^ mice, we generated WT, *Ptch1*^+/^^−^, and *Abhd17a*^−/−^*;Ptch1*^+/^^−^ mice for the following experiments. Western and qRT-PCR analyzes of cerebellar tissues demonstrated that *Ptch1* heterozygosity significantly increased both the protein levels of GLI1 and the mRNA levels of *Hhip* and *Ccnd2*, target genes of SHH signaling. Additional *Abhd17a* KO further enhanced GLI1 protein expression and transcriptional activation of these target genes in *Ptch1*^+/^^−^ mice (Fig. 8a, b), supporting the role of ABHD17A as a negative regulator of GLI1 stability and SHH signaling.

Phenotypic assessment revealed a significant increase in cerebellum-to-body weight ratios in *Ptch1*^+/^^−^ mice as compared to in WT controls and a further increase in *Abhd17a*^−/−^*;Ptch1*^+/^^−^ mice as compared to *Ptch1*^+/^^−^ mice (Fig. 8d). H&E staining of cerebellar sections at 3 months of age showed the normal cerebellar architecture in WT mice, whereas 3/8 of *Ptch1*^+/^^−^ mice developed mild MB_SHH_ characterized by small nodules of hyperproliferative GNPs in the EGL (black arrows, Fig. 8c). In contrast, 4/8 of *Abhd17a*^−/−^*;Ptch1*^+/^^−^ mice developed MB_SHH_ and 2/8 of them exhibited severe MB_SHH_ with tumor invasion up to half or entire folia (Fig. 8c). Although all mice with different genotypes maintained ten cerebellar folia (Fig. 8e), the foliar thickness was apparently reduced in *Abhd17a*^−/−^*;Ptch1*^+/^^−^ mice due primarily to the extensive invasion of tumors.

Immunofluorescence analysis revealed a significant increase in ectopic NeuN⁺ and BLBP⁺ cells in *Ptch1*^+/^^−^ mice as compared to in WT controls and a further increase in *Abhd17a*^−/−^*;Ptch1*^+/^^−^ mice as compared to in *Ptch1*^+/^^−^ mice. Calbindin⁺ PCs retained well-organized in the PCL across all groups (Supplementary Fig. 6e–h), suggesting that *Abhd17a* deletion exacerbates the phenotypes of *Ptch1* deficiency-induced MB_SHH_ at 3 months of age. At this age, the EGL is largely absent; consequently, very few cleaved caspase‑3⁺ cells were detected in this region. In *Ptch1*^+/^^−^ mice, however, EGL expansion led to a significant increase in the number of cleaved caspase-3⁺ cells compared to WT controls, whereas no significant difference was observed between *Abhd17a*^−/−^*;Ptch1*^+/^^−^ and *Ptch1*^+/^^−^ mice (Fig. 8f, i). Immunohistochemistry analysis further demonstrated that *Ptch1* heterozygosity increased both the number of PCNA⁺ cells and GLI1-derived immunosignals, both of which were further elevated upon *Abhd17a* knockout (Fig. 8g, h, j, k). Collectively, *Abhd17a* deletion amplifies SHH signaling activity, promotes tumor cell proliferation, and thereby aggravates the pathological severity of MB_SHH_ in *Ptch1*^+/^^−^ mice at 3 months of age. However, cross-sectional analysis at a single time point cannot distinguish whether this aggravated phenotype results from accelerated tumor initiation or enhanced tumor progression.

To address this question, we performed a longitudinal cohort study. A large cohort of *Ptch1*^+/^^−^ and *Abhd17a*^*−/−*^*;Ptch1*^*+/*^^*−*^ mice was generated by genetic crossing. These mice were closely monitored, and for each animal we recorded the time of first tumor appearance (indicated by a palpable bulge in the occipital region, as well as behavioral phenotypes such as ataxia and head tilt), the time of death, and the cause of death. Analysis of tumor incidence across all ages revealed an overall incidence of 34.9% (15/43) in *Ptch1*^+/^^−^ mice and 50% (11/22) in *Abhd17a*^*−/−*^*;Ptch1*^*+/*^^*−*^ mice, though this difference was not statistically significant (Fig. 8l). However, the incidence of MB_SHH_ within the first 3 months of age was significantly higher in *Abhd17a*^*−/−*^*;Ptch1*^*+/*^^−^ mice (45.5%, 10/22) compared with *Ptch1*^+/^^−^ mice (18.6%, 8/43) (Fig. 8m). These findings indicate that *Abhd17a* knockout promotes MB_SHH_ formation in *Ptch1*^+/^^−^ mice. This conclusion was further supported by tumor-free survival analysis, which showed that *Abhd17a* knockout reduced the median tumor-free survival from 197 days to 84 days (Fig. 8n). To evaluate the effect on tumor progression, we analyzed post-diagnosis survival of mice that had developed MB_SHH_. No significant difference in median post-diagnosis survival was observed between *Ptch1*^+/^^−^ and *Abhd17a*^*−/−*^*;Ptch1*^*+/−*^ mice, indicating that *Abhd17a* knockout does not affect tumor progression (Fig. 8o). Taken together, *Abhd17a* knockout increases the incidence of MB_SHH_ without influencing tumor progression.

## Discussion

By using biochemical, genetic, and clinical specimen approaches, we have for the first time uncovered the critical role of dynamic GLI1 palmitoylation in regulating SHH signaling and the incidence and progression of MB_SHH_. In this molecular event, ZDHHC13 catalyzes S-palmitoylation of GLI1 at Cys1034 to stabilize the protein by blocking its ubiquitination-dependent proteasomal degradation, whereas the depalmitoylase ABHD17A reverses this modification to attenuate SHH signaling. As a result, *Zdhhc13* loss-of-function robustly alleviates the severity of constitutively active SMO-induced MB_SHH_ and extends mouse survival_,_ while *Abhd17a* loss-of-function increases the incidence of MB_SHH_ in *Ptch1*^*+/*^^*−*^ mice.

Analysis of RNA-seq data from the GEO database identified a distinct expression profile of *DHHC* family genes in MB_SHH_. Among the 23 genes analyzed, *ZDHHC13* was significantly upregulated, consistent with immunohistochemistry findings in human MB_SHH_ specimens. We further demonstrated that ZDHHC13 overexpression activates the SHH pathway and contributes to MB_SHH_ pathogenesis, although the molecular drivers of its upregulation remain unknown. In the same transcriptomic dataset, *ZDHHC22* emerged as the most significantly downregulated *DHHC* gene, yet its functional relevance in MB_SHH_ is still unclear. Notably, although ABHD17A expression was not significantly altered in RNA-seq data or in a limited set of human clinical samples, the *GFAP-Cre;SMO-M2* cerebella did reveal a marked downregulation of *Abhd17a* in the tumor tissues. Functional experiments confirmed that *Abhd17a* knockout activates SHH signaling and increases tumor incidence, though the mechanism underlying its suppression in murine MB_SHH_ remains to be elucidated. Together, these findings highlight the ZDHHC13-ABHD17A regulatory circuit as essential for cerebellar homeostasis, whose disruption constitutes a critical driver of MB_SHH_ incidence and progression.

The current understanding of the crosstalk between S-palmitoylation and ubiquitination encompasses three predominant mechanisms: competitive binding between palmitoyltransferases and E3 ligases [33], enhanced E3 ligase accessibility following depalmitoylation [50], and altered ubiquitination status through membrane domain redistribution [51]. GLI1 contains two distinct degradation signal domains: D_N_ and D_C_, with the latter being recognized by the β-TrCP ubiquitin ligase complex [52]. Additionally, Numb facilitates the interaction between GLI1 and ITCH, promoting ubiquitination and degradation via two PPXY motifs and phospho-serine/proline motifs in the GLI1 C-terminal region [53]. Despite these insights, the E3 ligase complex specifically targeting depalmitoylated GLI1 remains unknown. We have identified that the Cys1034 residue resides within the C-terminal transcriptional activation domain of GLI1, which forms a charged flexible intrinsically disordered region (IDR). Such regions lack a stable three-dimensional structure, rendering side-chain amino acids, including lysines, fully exposed and thereby providing ideal accessibility for E3 ubiquitin ligase recognition and binding. It is noteworthy that the known D_N_ and D_C_ degrons of GLI1 also reside within IDRs. Based on these observations, we propose a novel regulatory model in which S-palmitoylation at Cys1034 may directly interfere through steric hindrance with the recognition and binding of a previously uncharacterized proximal degron by an E3 ligase, thereby stabilizing GLI1 and inhibiting its ubiquitin-proteasome-mediated degradation.

In canonical SHH signaling, primary cilia coordinate GLI activation through regulated protein trafficking. Upon SHH binding, dissociation of the SUFU-GLI complex enables GLI accumulation at the ciliary tip [54], facilitating its nuclear translocation and transcriptional activation of target genes [55, 56]. Our finding that ZDHHC13 stabilizes GLI1 via S-palmitoylation leads us to speculate that this modification may direct GLI1 to the ciliary tip, a mechanism that could explain how GLI1 evades degradation. Furthermore, ABHD17A itself requires S-palmitoylation for membrane localization and deacylase activity. Immunofluorescence reveals colocalization of GLI1 and ABHD17A near the plasma membrane, although their precise site of interaction remains unclear. We propose two potential regulatory mechanisms: (1) ciliary membrane-localized ABHD17A depalmitoylates GLI1 during its tip accumulation, or (2) an extra-ciliary interaction occurs following SUFU dissociation. Future live-cell imaging of GLI1 trafficking dynamics will help clarify these spatial regulatory mechanisms.

Immunofluorescence analysis revealed that GLI1 localizes predominantly to the PCL and EGL, with strong expression in the PCL. Both ZDHHC13 and ABHD17A co-localized with GLI1 in these regions, with ZDHHC13 showing more prominent expression in the PCL and ABHD17A distributed uniformly across layers. However, the developmental dynamics of ZDHHC13 and ABHD17A expression remain uncharacterized, and further investigation is warranted to determine the stage at which aberrant dynamic palmitoylation of GLI1 triggers MB_SHH_ pathogenesis. We utilized two transgenic mouse models that spontaneously develop MB_SHH_, *GFAP-Cre;Smo-M2* and *Ptch1*^*+/*^^*−*^, and confirmed elevated GLI1 expression and SHH pathway activity in the cerebella of both, thereby validating their relevance for MB_SHH_ studies. The *GFAP-Cre;Smo-M2* model exhibits nearly 100% penetrance, with severe tumor pathology typically observable by P14. In our experiments, excessive proliferation of GNPs and disrupted foliation were observed as early as P4, suggesting that SMO activation influences GNP behavior before this stage, possibly during embryonic development prior to the first wave of GNP proliferation around E13.5-E15.5. This early dysregulation may explain the 100% MB_SHH_ incidence observed in both *Zdhhc13*^*f/f*^, *GFAP-Cre;Zdhhc13*^*f/f*^ and *GFAP-Cre;Smo-M2* mice. We hypothesize that *Zdhhc13* expression begins after the initial *SMO-M2*-driven proliferative phase, and thus its knockout may attenuate only later stages of disease progression rather than preventing tumor initiation. In contrast to the early-onset, high-penetrance phenotype of the *GFAP-Cre;Smo-M2* model, *Ptch1*^*+/*^^*−*^ mice typically develop MB_SHH_ between 3 and 6 months of age with only 15-20% penetrance. Homozygous *Ptch1*^*−/−*^ mutants are embryonically lethal due to severe neural tube defects. Notably, the histopathological features of MB_SHH_ in *Ptch1*^+/^^−^ mice closely mirror those of human MB_SHH_ [49], underscoring the translational relevance of this model. Unlike the *GFAP-Cre;Smo-M2* model, in which MB_SHH_ develops with 100% penetrance, *Ptch1*^*+/*^^*−*^ mice harbor a heterozygous deletion and require a second hit, such as loss of heterozygosity (LOH) or additional signaling accumulation, to initiate tumorigenesis [57]. We propose that *Abhd17a* knockout further activates SHH signaling, thereby promoting signal accumulation that enables *Ptch1*^*+/*^^*−*^ mice to cross the tumorigenic threshold, leading to earlier MB_SHH_ onset. Once this threshold is crossed, however, the effect conferred by *Abhd17a* knockout may be insufficient to influence tumor progression, consistent with our finding that *Abhd17a* knockout does not affect post-diagnosis survival. Future studies with larger cohorts will be valuable to further validate this concept. This mechanistic distinction explains why *Zdhhc13* knockout affects the progression of SMO-driven MB_SHH_, whereas *Abhd17a* knockout promotes MB_SHH_ formation in *Ptch1*^*+/*^^*−*^ mice without affecting its progression.

In summary, the present study provides the first evidence for dynamic S-palmitoylation of GLI1, a key transcription factor in the SHH pathway. The ZDHHC13-mediated S-palmitoylation stabilizes GLI1, while ABHD17A-mediated depalmitoylation promotes its degradation, forming a dynamic equilibrium that precisely regulates both GLI1 stability and SHH transcriptional output. Both ZDHHC13 gain-of-function and ABHD17A loss-of-function contribute to the activation of SHH signaling and, respectively, drive the progression or incidence of MB_SHH_. Interventions targeting this dynamic modification may provide an additional target for the therapy of SHH-driven malignancies, such as MB and BCC.

## Materials and methods

### Clinical specimens

Paraffin-embedded tissue sections and corresponding medical records from pediatric patients with a pathological diagnosis of SHH-MB and Group 3/4 MB were obtained from the Department of Pathology, Children’s Hospital of Zhejiang University School of Medicine, and used for immunohistochemistry staining. This retrospective study was approved by the Ethics Committee of the Children’s Hospital of Zhejiang University School of Medicine (Approval No. 2023-IRB-0233-P-01). In addition, a commercially available medulloblastoma tissue microarray (No. N035Cb01; Bioaitech Co., Ltd, Xi’an, China) was used for immunohistochemistry staining followed by correlation analysis.

### Mouse strains

The constitutively active form of SMO (*Smo-M2)-*overexpressing mouse strain was purchased from the Jackson Laboratory (Bar Harbor, ME). The *Zdhhc13*^*flox/+*^, *Abhd17a*^*+/*^^*−*^, and *Ptch1*^*+/*^^*−*^ mouse strains were purchased from Cyagen Biosciences (Suzhou, China). *GFAP-Cre* mice were gifted from Professor Chong Liu at Zhejiang University School of Medicine. All animals were housed and bred under specific pathogen-free (SPF) conditions in the Hangzhou City University Animal Care Facility, in accordance with institutional guidelines for laboratory animal care. For all in vivo experiments, mice were grouped by genotype without randomization, no blinding was performed, and sample sizes were based on previous experiments and accounted for biological/technical variability.

### Cell culture, transfection and lentivirus infection

Human embryonic kidney 293T (HEK293T) and MB_SHH_ Daoy cells were purchased from the American Type Culture Collection (ATCC, Manassas, VA). NIH3T3 cells were purchased from the National Collection of Authenticated Cell Cultures (Shanghai, China). All cell lines were cultured in high-glucose Dulbecco’s Modified Eagle Medium (DMEM) supplemented with 10% fetal bovine serum (FBS, Gibco, Grand Island, NY) and 1% penicillin-streptomycin (Sigma-Aldrich, St. Louis, MO), and incubated at 37 °C in a humidified atmosphere containing 5% CO₂. Transient transfections were performed by using Lipofectamine 2000 (Invitrogen, Carlsbad, CA) according to the manufacturer’s instructions. For lentivirus infection, the target gene constructs (in the pCDH vector) were co-transfected with the packaging plasmids Vsvg, pMDL, and Rev into HEK293T cells in a 10-cm dish at a ratio of 2:1:1:1. The viral supernatant was harvested 48 h post transfection and filtered through a 0.45 µm membrane. Concurrently, Daoy cells were seeded and infected at approximately 60% confluence by replacing half of the culture medium with the filtered viral supernatant, supplemented with 10 µg/mL polybrene. This infection process was repeated every 12 h until fluorescence was visible in the target cells. Infection efficiency was validated by qRT-PCR.

### Antibodies, reagents and chemicals

The antibodies used were purchased from: DYKDDDDK (RA1003-02, Vazyme, Nanjing, China), HA tag (RA1004-00, Vazyme), Myc tag (RA1005-00, Vazyme), ABHD17A (K110691P, Solarbio, Beijing, China), ABHD17A (K110690P, Solarbio), ZDHHC13 (ab28759, Abcam, Cambridge, UK), BLBP (ab32423, Abcam), NeuN (ET1602-12, HUABIO, Hangzhou, China), Calbindin (ET1702-54, HUABIO), Alpha tubulin (HA721914, HUABIO), GAPDH (ET1601-4, HUABIO), GLI1 (ET1702-85, HUABIO), GLI1 (66905-1-Ig, Proteintech, Wuhan, China), PCNA (ET1605-38, HUABIO), cleaved caspase-3 (25128-1-AP, Proteintech), IRDye 680RD Goat anti-Mouse (926-68070, LI-COR, Lincoln, NE), IRDye 800CW Goat anti-Rabbit (926-32211, LI-COR), Alexa Fluor 568 goat anti-mouse (A-11004, Invitrogen), Alexa Fluor 488 goat anti-rabbit (A-11008, Introvigen). Magnetic beads for immunoprecipitation were purchased from Selleck (Houston, TX): Anti-DYKDDDDK (Flag) magnetic beads (B26102, Selleck), Anti-Myc magnetic beads (B26302, Selleck), Protein A/G magnetic beads (B23202, Selleck). Chemicals were purchased from: Cycloheximide (HY-12320, MCE), Actinomycin D (HY-17559, MCE), MG-132 (HY-13259, MCE), Bafilomycin A1 (HY-100558, MCE), Chloroquine (HY-17589A, MCE), 2-Bromohexadecanoic acid (238422, Sigma-Aldrich), N-Ethylmaleimide (E1271, Sigma-Aldrich), and Hydroxylamine hydrochloride (ST093, Beyotime, Shanghai, China).

### Plasmids construction

The following plasmids were constructed by cloning into the pXJ-40 vector with the indicated tags: Flag-GLI1, Myc-GLI1, Myc-GLI3, HA-ZDHHC13, Flag-APT1, Flag-APT2, Myc-ABHD17A, Flag-ABHD17A/B, HA-Gli1 (mouse) and Myc-Abhd17a (mouse). The HA-GLI1, Gli-luciferase reporter and Renilla plasmids were kindly provided by Dr. Fanxin Long (University of Washington). Plasmids encoding HA-ubiquitin, Flag-Zdhhc13 (mouse), Flag-ABHD17C (human), and Flag-GLI2 (human) were purchased from Miaolingbio (Wuhan, China). For lentiviral knockdown, shRNAs targeting *ZDHHC13* or *ABHD17A* were cloned into the pSicoR-GFP vector. The targeting sequences were as follows: Sh1-ZDHHC13: CTCATGTTATCAGCTCACAAA; Sh2-ZDHHC13: TACTCACTGATTATGGATAAA; Sh3-ZDHHC13: CATTTACCTATGGTCATATTA; sh1-ABHD17A: AGGTGTCCAAGATCACGTCTC; sh2-ABHD17A: GCCTGCGGGTACATCCACCTT; Sh3-ABHD17A: CCAGATGAGCAGCTTCTACAT. For overexpression, the pCDH-CMV-MCS-3 × Flag-Puro vector (Miaolingbio) was used to generate lentiviral constructs expressing ZDHHC13 or ABHD17A. Target gene fragments were amplified using the KOD-Plus-Neo kit (KOD-401, TOYOBO, Osaka, Japan), and site-directed mutagenesis was performed with the KOD-Plus Mutagenesis Kit (SMK-101, TOYOBO). All primers were synthesized and all constructs verified by DNA sequencing at Youkang Biology (Hangzhou, China).

### Protein extraction, immunoprecipitation and western blotting

For protein extraction, adherent cells were washed twice with cold PBS and lysed on ice for 30 min using RIPA lysis buffer (P0013C, Beyotime) containing protease and phosphatase inhibitors (B14001, B15001-A/B, Selleck). Lysates were centrifuged at 13,000 rpm for 15 min at 4 °C, and the supernatant was collected for protein quantification with a BCA assay kit (P0011, Beyotime). After normalization, protein samples were mixed with loading buffer and denatured at 100 °C for 5 min.

For co-immunoprecipitation (Co-IP), target proteins were captured using anti-Flag/Myc/HA magnetic beads or antibody-conjugated Protein A/G magnetic beads overnight at 4 °C with rotation. Beads were washed three times with IP lysis buffer (P0013, Beyotime), and bound proteins were eluted in 2× loading buffer by boiling at 100 °C for 10 min. Eluates were analyzed immediately or stored at −80 °C.

For western blotting, protein samples were separated by 8–15% SDS-PAGE and transferred to PVDF membranes. After blocking with non-fat milk or BSA (A8020, Solarbio), membranes were incubated with primary and corresponding secondary antibodies. Signal detection was performed using an Odyssey Infrared Imaging System (LI-COR), and band intensities were quantified with ImageJ software. GAPDH and α-tubulin served as loading controls. The antibodies used in this study were as follows: DYKDDDDK (1:2000, RA1003-02, Vazyme), HA tag (1:1000, RA1004-00, Vazyme), Myc tag (1:1000, RA1005-00, Vazyme), ABHD17A (1:1000, K110690P, Solarbio), ZDHHC13 (1:1000, ab28759, Abcam), α-tubulin (1:5000, HA721914, HUABIO), GAPDH (1:3000, ET1601-4, HUABIO), GLI1 (1:500, ET1702-85, HUABIO), IRDye 680RD Goat anti-Mouse (1:10,000, 926-68070, LI-COR), IRDye 800CW Goat anti-Rabbit (1:10,000, 926-32211, LI-COR).

### Acyl-biotin exchange (ABE) assay

Forty-eight hours post-transfection, cells were washed with cold PBS and lysed in NP-40 lysis buffer (pH 7.4, P0013F, Beyotime) containing protease inhibitors, phosphatase inhibitors A&B, and 50 mM NEM. After 30 min on ice and centrifugation (13,000 × *g*, 15 min, 4 °C), the supernatant was immunoprecipitated with anti-Flag magnetic beads overnight at 4 °C. Beads were washed with pH 7.4 lysis buffer (five times) and pH 7.2 buffer (three times), then split into -HAM (one-third) and +HAM (two-thirds) groups. The +HAM sample was incubated with 1 M hydroxylamine in pH 7.2 buffer, while the -HAM control received buffer only, for 1 h at room temperature. Beads were then washed with pH 7.2 (four times) and pH 6.2 (three times) buffers. Subsequently, beads were resuspended in pH 6.2 buffer and labeled with 5 μM Biotin-BMCC for 1 h at 4 °C. After five washes with pH 6.2 buffer, samples were eluted in loading buffer by boiling at 100 °C for 10 min. Proteins were analyzed by western blot using Streptavidin-HRP (1:1000, ab7403, Abcam). For ABE detection, membranes were blocked with 3–5% BSA to prevent endogenous biotin interference.

### RNA extraction and quantitative real-time PCR

Total RNA was extracted from cells or tissues using RNAiso Plus (9108, Takara, Shiga, Japan) according to the manufacturer’s instructions. Subsequently, 2 μg of total RNA was reverse-transcribed into complementary DNA (cDNA) using the 5× HiScript II qRT Supermix (R222-01, Vazyme). Quantitative real-time PCR was performed with the 2× ChamQ Universal SYBR qPCR Master Mix (Q711-02, Vazyme) to determine the mRNA expression levels of target genes. The expression of target genes was normalized to the endogenous control β-actin, and relative fold changes in mRNA levels were calculated using the 2^^-ΔΔCt^ method. The sequences of primers used are listed in Supplementary table 1.

### Dual-luciferase reporter assays

HEK293T cells were seeded in 24-well plates and co-transfected with 0.5 μg of HA-GLI1, 0.5 μg of Gli-luciferase reporter, and 0.05 μg of Renilla control plasmids per well. Forty-eight hours after transfection, cells were lysed and luciferase activity was measured using a dual-luciferase reporter assay kit (E1960, Promega, Madison, WI) according to the manufacturer’s instructions. Renilla luciferase activity served as an internal standard. All experiments were performed in three independent replicates, with each replicate including triplicate wells.

### Immunofluorescence and immunohistochemistry

Paraffin-embedded cerebellar tissues were sectioned at a thickness of 4 μm. For immunohistochemistry (IHC), sections were processed using a Mouse or Rabbit Two-Step Assay Kit (PV-6002/PV-6001, ZSGB-BIO) according to the manufacturer’s protocol. Primary antibodies against GLI1 (1:50, ET1702-85, HUABIO) and PCNA (1:1000, ET1605-38, HUABIO) were applied and incubated overnight at 4 °C. Immunoreactivity was visualized with a DAB substrate kit (DA1010, Solarbio). Quantification of the staining intensity was performed using the Average Optical Density (AOD), defined as the Integrated Optical Density (IOD) per unit area, to normalize for variations in the positive area. For immunofluorescence (IF), sections were permeabilized with 0.3% Triton X-100 in PBS for 30 min, followed by three washes with PBS. Non-specific binding was blocked with 3% bovine serum albumin (BSA) for 1 h at room temperature. Sections were then incubated overnight at 4 °C with the following primary antibodies: GLI1 (1:50, ET1702-85, HUABIO), ABHD17A (1:100, K110691P, Solarbio), ZDHHC13 (1:50, ab28759, Abcam), BLBP (1:50, ab32423, Abcam), NeuN (1:100, ET1602-12, HUABIO), Calbindin (1:100, ET1702-54, HUABIO), and cleaved caspase-3 (1:500, 25128-1-AP, Proteintech). After three washes, the sections were incubated with Alexa Fluor 568- or Alexa Fluor 488-conjugated secondary antibodies (1:1000, A-11004/A-11008, Invitrogen) at 37 °C for 1 h. Nuclei were counterstained with DAPI, and slides were mounted with an anti-fluorescence quenching agent (E-IR-R119, Elabscience, Wuhan, China). All images were acquired with an Olympus FV3000 confocal microscope and analyzed quantitatively using ImageJ software.

### MnCl_2_ enhanced magnetic resonance imaging

On postnatal day 14, mice received an intraperitoneal injection of 15 mM manganese chloride (MnCl_2_, 221279, Sigma) dissolved in physiological saline (0.9% NaCl), administered 24 h prior to magnetic resonance imaging (MRI). The injection volume was calculated as 0.13 mL per 10 g body weight, corresponding to a final dose of 0.2 mmol/kg (equivalent to 40 mg/kg body weight). No adverse effects attributable to MnCl₂ administration were observed at this dosage. The chosen dose was based on previously validated manganese-enhanced magnetic resonance imaging (MEMRI) protocols for brain imaging [58]. MRI was performed using a Pharmascan 7T preclinical system (Bruker, Billerica, Germany) with a 7 Tesla, 16 cm bore magnet, a 90 mm inner diameter gradient coil, a gradient strength of 570 mT/m, and a slew rate of 5130 T/m/s. The system includes an actively shielded gradient set and integrated shim coils to ensure optimal magnetic field homogeneity. Mice were anesthetized with isoflurane (induction at 3–4%, maintenance at 0.5–1.5% in air). T1-weighted images were acquired in both sagittal and coronal orientations. Sagittal imaging parameters were: Echo Time (TE) = 6 ms; Repetition Time (TR) = 800 ms; Number of averages = 6; Repetitions = 1; Slices = 26; Slice thickness (ST) = 0.4 mm; Field of view (FOV) = 14 mm × 12 mm; image size = 256 mm × 218 mm. Coronal imaging parameters were: TE = 6 ms; TR = 800 ms; Number of averages = 6; Repetitions = 1; Slices = 20; ST = 0.4 mm; FOV = 14 mm × 14 mm; image size = 256 mm × 256 mm. The imaging procedure for each mouse required 20 min. Quantitative analysis of lateral ventricle volume was performed on T1-weighted two-dimensional (2D) MRI sequences using semi-automated segmentation and volumetric calculation on 2D slices with 3D Slicer version 5.10.0 (Boston, MA). The final comparison was based on the ratio of lateral ventricle volume to total intracranial volume (TIV).

### Statistical analysis

All numerical data were presented as mean ± SD. Statistical analyzes and graphing were performed using GraphPad Prism version 8.2.1 (GraphPad Software, San Diego, CA). For comparisons among multiple groups, one-way analysis of variance (ANOVA) was applied, followed by Tukey-Kramer post hoc testing. Comparisons between two groups were conducted using Student’s *t*-test. Survival analysis was performed using the log-rank test. Comparisons of the incidence were performed using Fisher’s exact test. Statistical significance was assessed at *p* < 0.05, *p* < 0.01. All experiments were independently repeated at least three times with consistent results, and representative data are shown in the figures.

## Supplementary information

Supplementary figures and table

Original Western Blot

## Data Availability

The RNA-Seq data utilized within this study were retrieved from the Gene Expression Omnibus (GEO) database, specifically the series accessioned as GSE85217. The link to access the dataset is: https://www.ncbi.nlm.nih.gov/geo/query/acc.cgi?acc=GSE85217. All data underpinning the findings of this research are comprehensively presented within the main text and supplementary information files.
