# Supplementary material for: Dynamic S-palmitoylation of glioma-associated oncogene 1 regulates the incidence and progression of Sonic Hedgehog medulloblastoma
Source: Cell Death Dis. 2026 Jun 29;17(1):946. doi: 10.1038/s41419-026-09051-y (PMC13743765; doi:10.1038/s41419-026-09051-y)

Legends for Supplementary figures:

Supplementary figure 1: 2-BP inactivates SHH signaling by inhibiting the S-palmitoylation of GLI1. (a) Western analyses of exogenous GLI1 protein in HA-GLI1-expressing HEK293T cells or endogenous GLI1 protein in Daoy cells, following treatment with 25 μM 2-BP for the indicated times. (b) Western analyses of exogenous GLI2 or GLI3 protein in HEK293T cells transfected with Flag-GLI2 or Myc-GLI3 construct and treated with 25 μM 2-BP for 24 h. (c) Western analyses of exogenous GLI1 protein in HA-GLI1-expressing HEK293T cells treated with or without 25 μM 2-BP for 24 h in combination with 5 μg/mL actinomycin D for the indicated times. (d) qRT-PCR analysis of *GLI1* mRNA levels in HEK293T cells treated with 25 μM 2-BP for the indicated times (n=3). Mean ± SD.

Supplementary figure 2: ZDHHC13 palmitoylates and stabilizes GLI1 to activate SHH signaling. (a) qRT-PCR analysis of *ZDHHC13* mRNA levels in HEK293T cells transfected with different sh-ZDHHC13 construct for 72 h (n=3). (b) qRT-PCR analysis of *ZDHHC13* mRNA levels in Daoy cells infected with lentiviruses expressing Flag-ZDHHC13 (n=3). (c) Western analyses of endogenous GLI1 protein in HEK293T cells transfected with different sh-ZDHHC13 constructs for 72 h. (d) Western analyses of exogenous GLI1 protein levels in HA-GLI1-expressing HEK293T cells transfected with or without Flag-ZDHHC13 construct, and then treated with 5 μg/mL actinomycin D for the indicated times. (e) Validation of *Zdhhc13* knockdown efficiency by qRT-PCR in cerebella from *Zdhhc13*$^{f/f}$ and *GFAP*-

Cre;Zdhhc13$^{f/f}$ pups (n=5). (f) Western analyses of GLI1 protein in cerebella of 2-week-old *Zdhhc13*$^{f/f}$ and *GFAP-Cre;Zdhhc13*$^{f/f}$ pups (n=3). Mean ± SD; one-way ANOVA with Tukey's multiple comparison test (a) or Student's t-test (b, e); $^{**}p<0.01$.

Supplementary figure 3: ABHD17A depalmitoylates and destabilizes GLI1 to inactivate SHH signaling. (a and b) Western analyses of exogenous GLI1 protein in HEK293T cells co-transfected with Myc-GLI1 and either Flag-APT1/2 or ABHD17A/B/C constructs. (c) qRT-PCR analysis of *ABHD17A* mRNA levels in Daoy cells infected with lentiviruses expressing Flag-ABHD17A (n=3). (d) qRT-PCR analysis of *ABHD17A* mRNA levels in HEK293T cells transfected with different sh-ABHD17A constructs for 72 h (n=3). (e) Western analyses of endogenous GLI1 protein in HEK293T cells transfected with different sh-ABHD17A constructs for 72 h. (f) Western analyses of exogenous GLI1 protein in Flag-GLI1-expressing HEK293T cells transfected with or without Myc-ABHD17A construct, and then treated with 5 μg/mL actinomycin D for the indicated times. (g) Validation of *Abhd17a* knockdown efficiency by qRT-PCR in cerebella from WT and *Abhd17a* KO mice (n=3). (h) Western analyses of GLI1 protein in cerebella of 2-week-old WT and *Abhd17a* KO pups (n=3). (i) qRT-PCR analysis of *ABHD17A* mRNA levels in cerebella of 2-week-old *SMO-M2* and *GFAP-Cre;SMO-M2* pups (n=3). Mean ± SD; one-way ANOVA with Tukey's multiple comparison test (d) or Student's t-test (c, g, i); $^{*}p<0.05$, $^{**}p<0.01$.

Supplementary figure 4: Cys1034 is responsible for ZDHHC13/ABHD17A-

induced human GLI1 palmitoylation/depalmitoylation. (a) Construction of GLI1 truncation mutants with different segment deletions. (b) Western analyses of Flag-tagged GLI1 truncation mutants expressed in HEK293T cells and treated with 25 μM 2-BP for 24 h.

Supplementary figure 5: *Zdhhc13* loss-of-function relieves the severity of MB_{SHH} induced by *SMO-M2* overexpression in mice. (a) Immunofluorescence analysis of co-localization of GLI1 with ZDHHC13 in the mouse cerebella at P4. Bar=20 μm. (b) Representative coronal MRI images from one mouse in each of the four groups are shown. Seven of the total 20 slices are presented, and the slice number is indicated above each corresponding image.

Supplementary figure 6: *Abhd17a* loss-of-function increases the incidence of MB_{SHH} in *Ptch1*$^{+/-}$ mice. (a) qRT-PCR analysis of *Ptch1* mRNA levels in cerebella from WT and *Abhd17a* KO mice at P14 (n=3). (b) The number of folia in the sagittal section of the cerebellar vermis (n=5). (c) H&E staining of cerebellar paraffin sections from WT and *Abhd17a* KO mice at P14 or 3 months of age. Bar=0.5 mm. (d) Immunofluorescence analysis of co-localization of GLI1 with ABHD17A in the mouse cerebella at P4. Bar=20 μm. (e-h) Anti-NeuN, anti-Calbindin and anti-BLBP immunofluorescence labeling and quantification results in the sagittal sections of the cerebellar vermis. Bar=20 μm; n=3. Mean ± SD; one-way ANOVA with Tukey’s multiple comparison test (f-h) or Student's t-test (a-b); ns: no significance, $^{*}p<0.05$.

Supplementary table 1: The primers used for qRT-PCR.

Mouse *Zdhhc13*-F	ACTGGGCTGCCATTAACAACA
Mouse *Zdhhc13*-R	GCAAATGCCCTTGTCGGATG
Mouse *Abhd17a*-F	CTGGAAGATCCACCTGACGG
Mouse *Abhd17a*-R	GCATTGCCATGCGAGAAGAG
Mouse *β-actin*-F	CACGATGGAGGGGCCGGACTCATC
Mouse *β-actin*-R	TAAAGACCTCTATGCCAACACAGT
Mouse *Gli1*-F	CCAAGCCAACTTTATGTCAGGG
Mouse *Gli1*-R	AGCCCGCTTCTTTGTTAATTTGA
Mouse *Ptch1*-F	AAAGAACTGCGGCAAGTTTTTG
Mouse *Ptch1*-F	CTTCTCCTATCTTCTGACGGGT
Human *β-actin*-F	GACGATGGAGGGGCCGGACTCGTC
Human *β-actin*-R	CAAAGACCTGTACGCCAACACAGT
Human *GLI1*-F	AACGCTATACAGATCCTAGCTCG
Human *GLI1*-R	GTGCCGTTTGGTCACATGG
Human *PTCH1*-F	CCAGAAAGTATATGCACTGGCA
Human *PTCH1*-R	GTGCTCGTACATTTGCTTGGG
Human *ZDHHC13*-F	GCGCCTCGAGATGGAGGGGCCGGGGCTGGGCT
Human *ZDHHC13*-R	GCGGGGTACCTCATACTGAGCGAAGAACCTTC
Human *ABHD17A*-F	TGCCGACTTCCAGTACAGC
Human *ABHD17A*-R	CACGCAGCGAACATACATGC

Supplementary figure 1

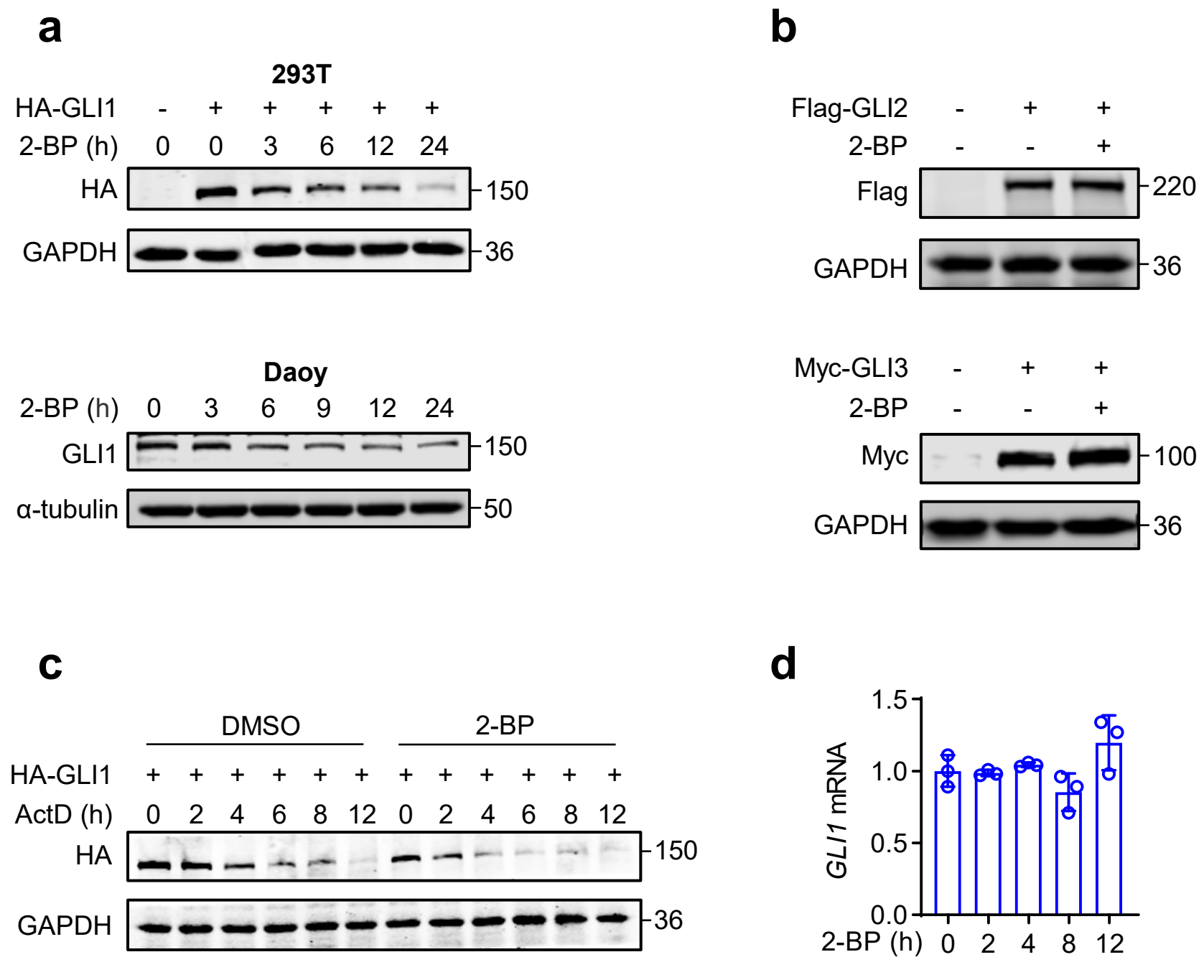

Supplementary figure 2

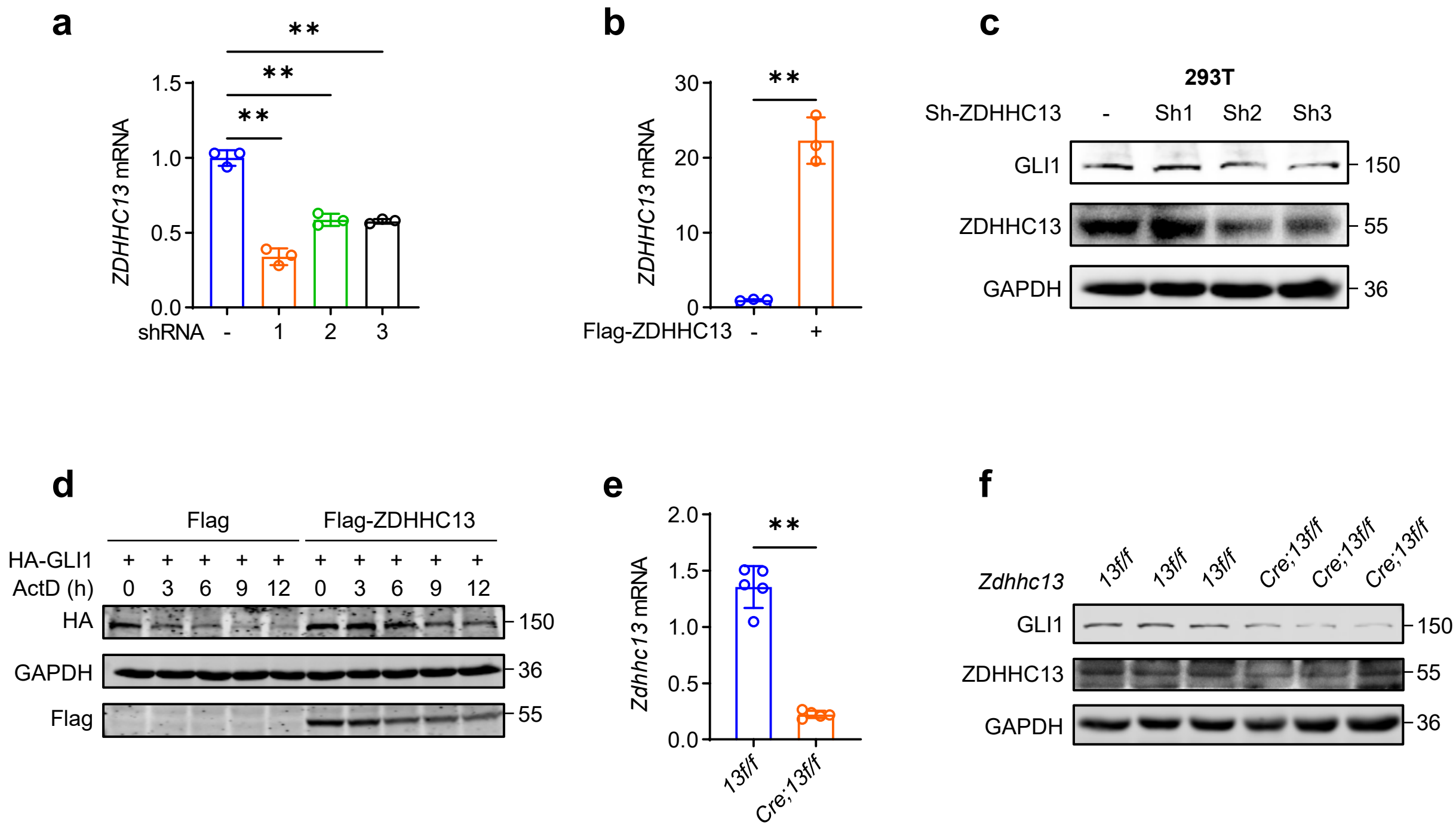

Supplementary figure 3

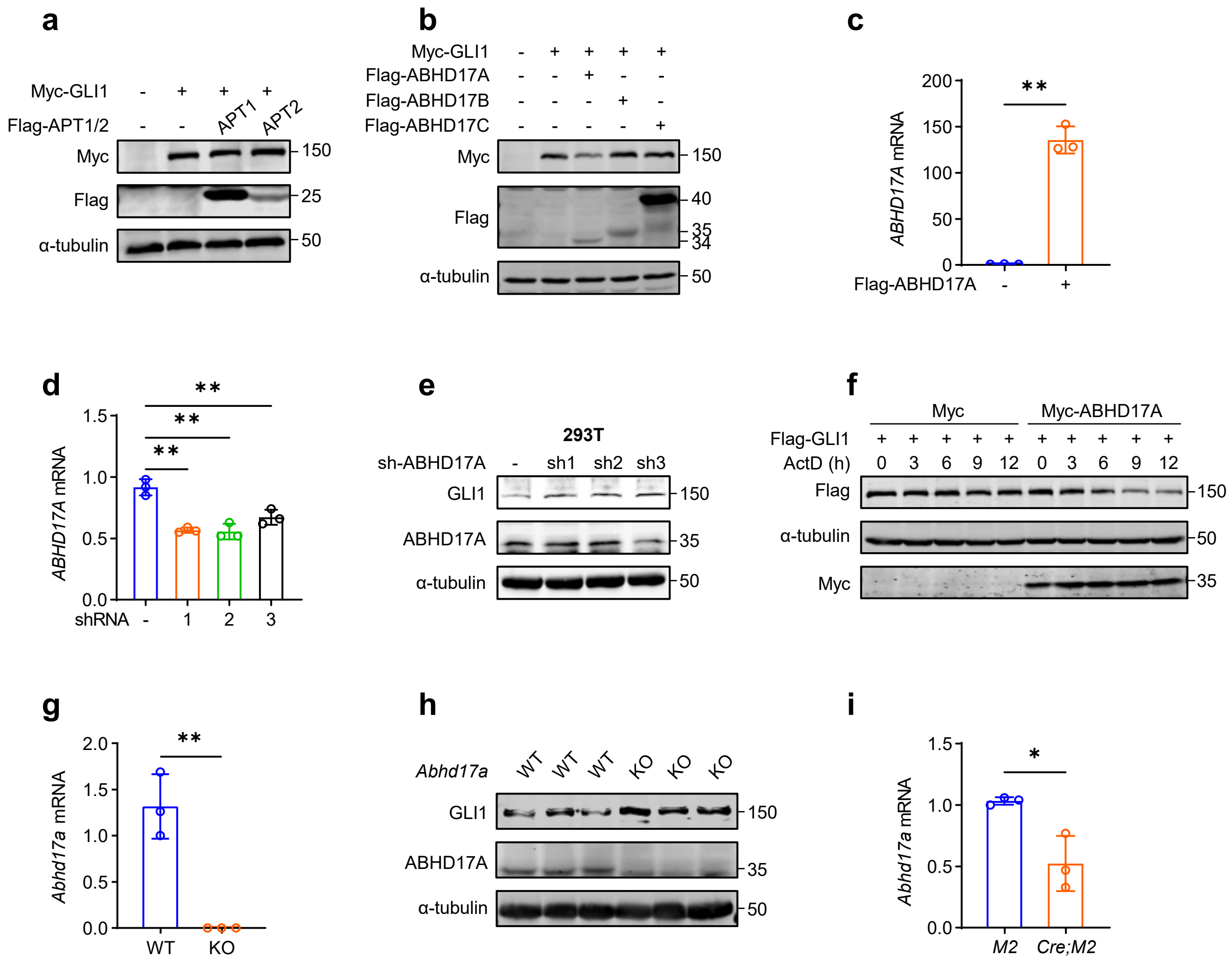

Supplementary figure 4

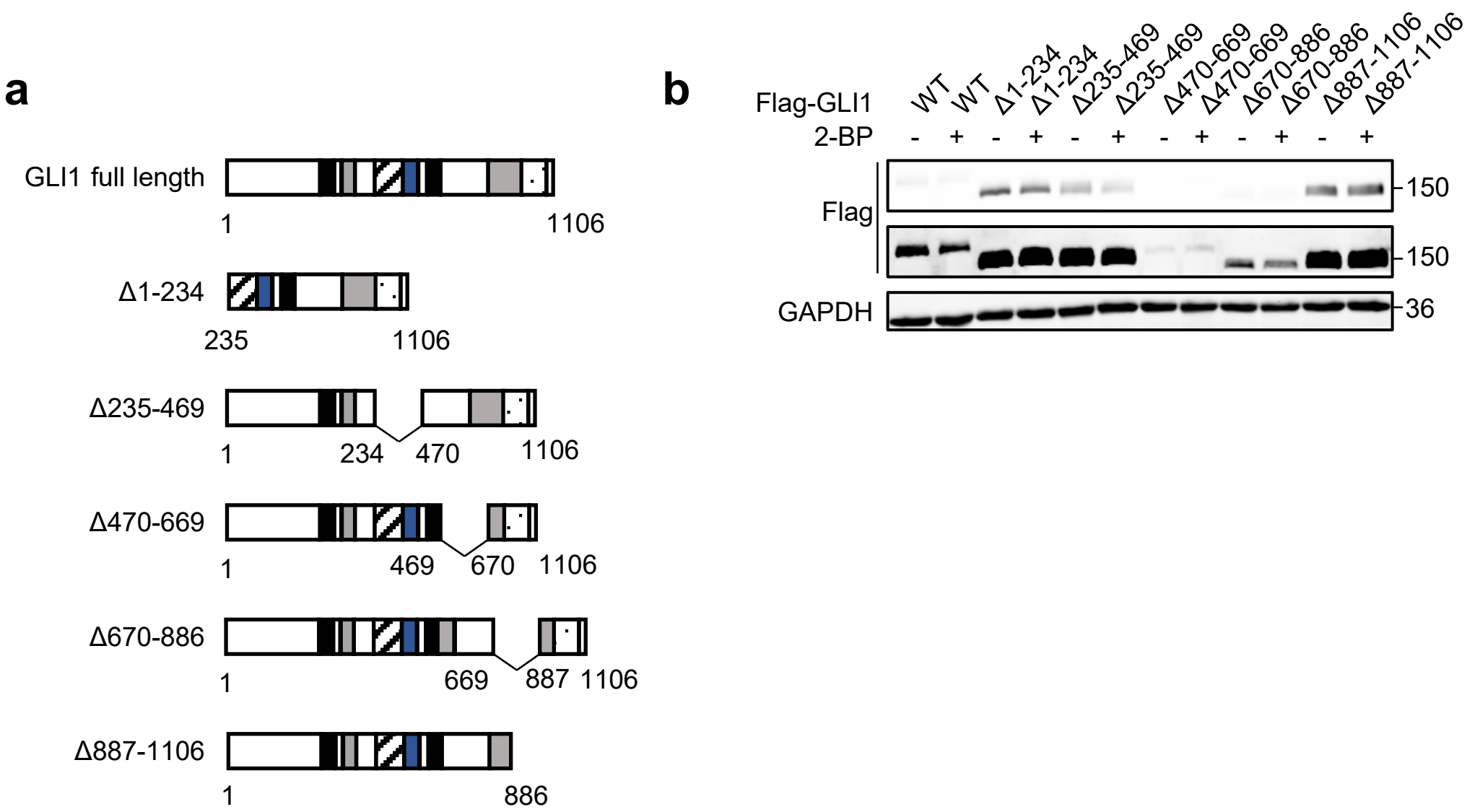

Supplementary figure 5

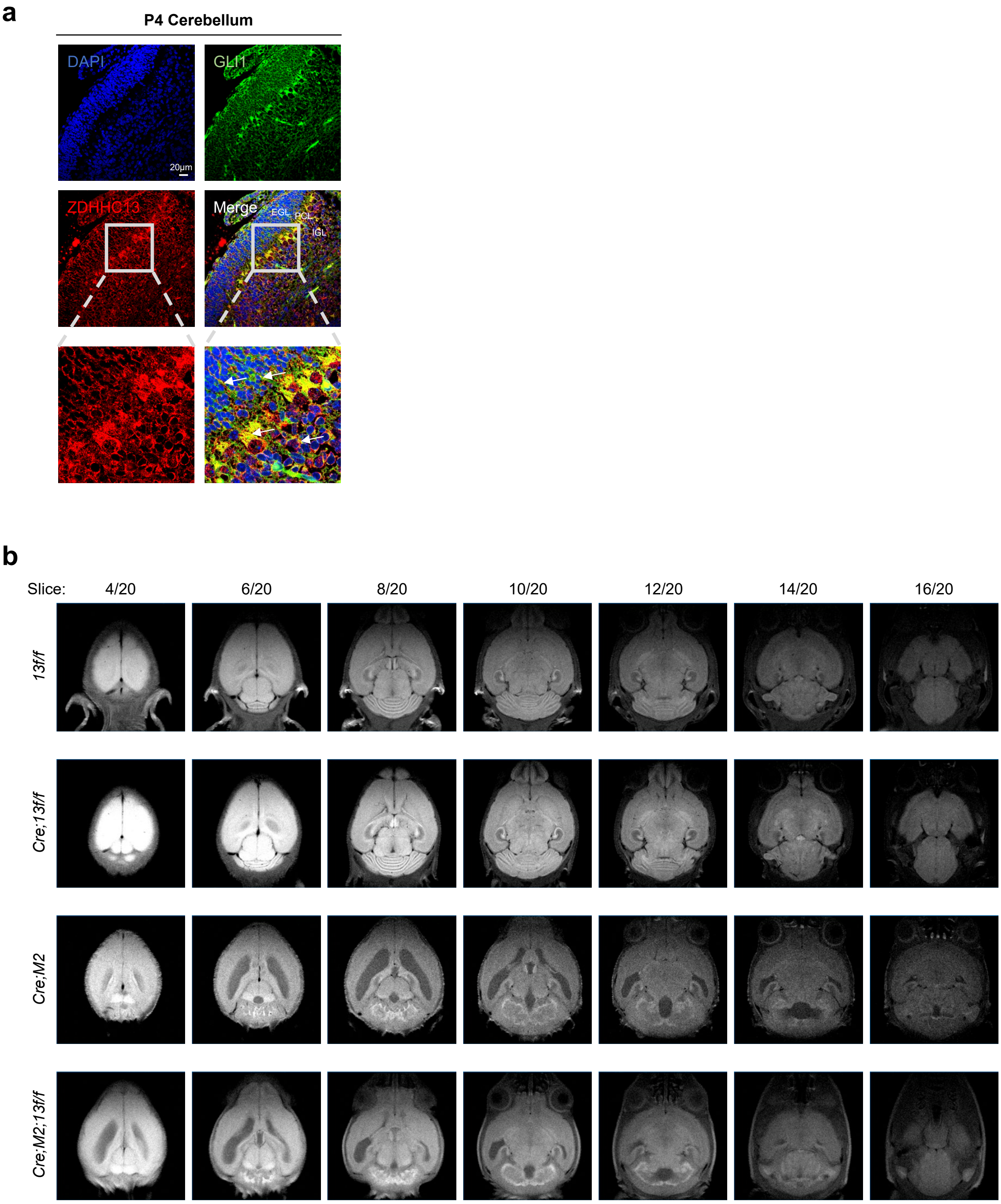

Supplementary figure 6

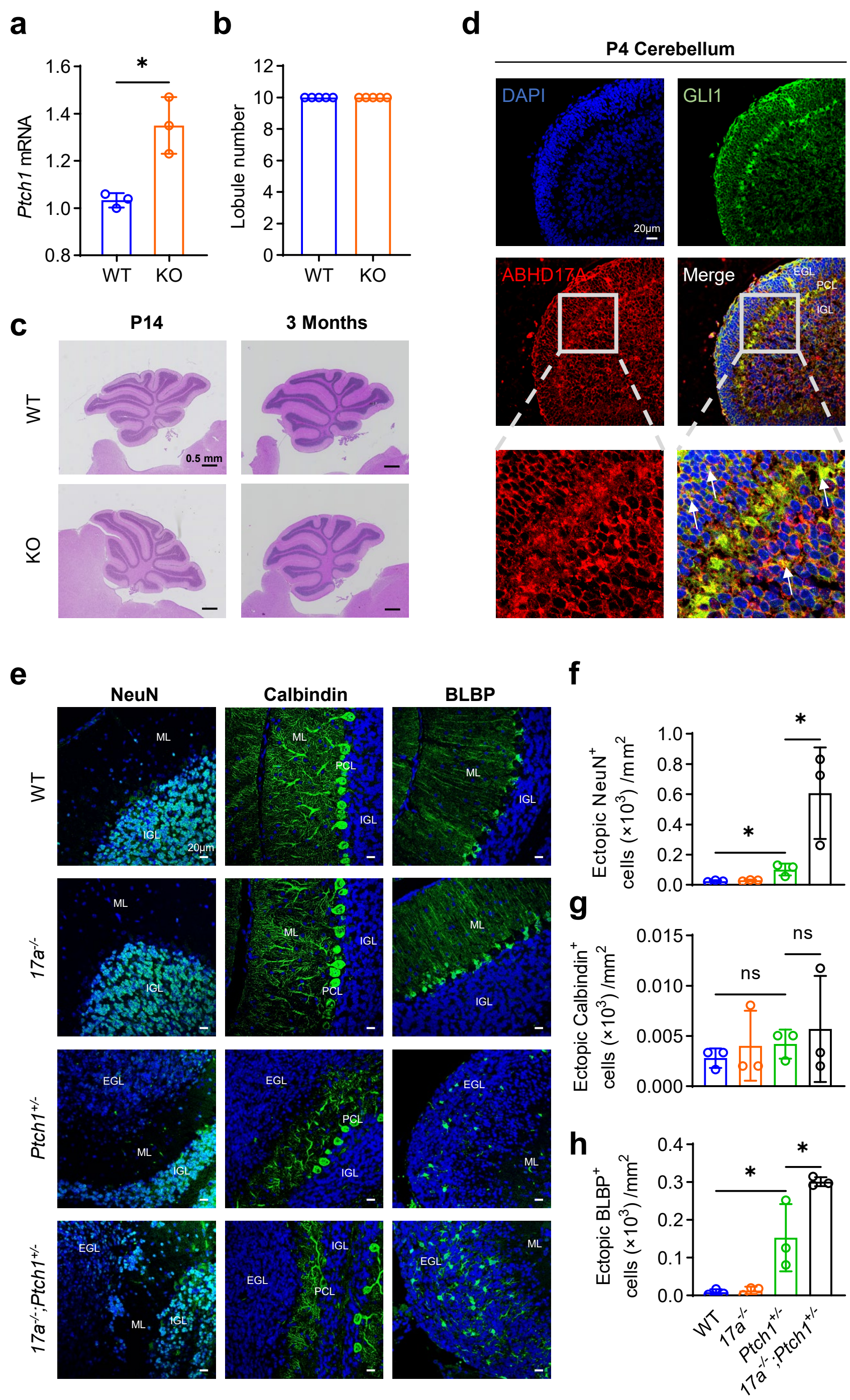

Supplement: Supplementary file 1 [file 41419_2026_9051_MOESM1_ESM.pdf]
